# Coding-Gene Coevolution Analysis of Rotavirus Proteins: A Bioinformatics and Statistical Approach

**DOI:** 10.3390/genes11010028

**Published:** 2019-12-24

**Authors:** Nabil Abid, Giovanni Chillemi, Marco Salemi

**Affiliations:** 1 Laboratory of Transmissible Diseases and Biological Active Substances LR99ES27, Faculty of Pharmacy, University of Monastir, Rue Ibn Sina, Monastir 5000, Tunisia; 2 High Institute of Biotechnology of Sidi Thabet, Department of Biotechnology, University Manouba, BP-66, Ariana-Tunis 2020, Tunisia; 3 Department for Innovation in Biological, Agro-food and Forest systems, DIBAF, University of Tuscia, via S. Camillo de Lellis s.n.c., 01100 Viterbo, Italy; gchillemi@unitus.it; 4 Institute of Biomembranes, Bioenergetics and Molecular Biotechnologies, IBIOM, CNR, Via Giovanni Amendola, 122/O, 70126 Bari, Italy; 5 Department of Pathology, Immunology and Laboratory Medicine, University of Florida College of Medicine, Emerging Pathogens Institute, P.O. Box 100009, Gainesville, FL 32610-3633, USA; salemi@pathology.ufl.edu

**Keywords:** rotavirus, bioinformatics, statistics

## Abstract

Rotavirus remains a major cause of diarrhea in infants and young children worldwide. The permanent emergence of new genotypes puts the potential effectiveness of vaccines under serious question. The distribution of unusual genotypes subject to viral fitness is influenced by interactions among viral proteins. The present work aimed at analyzing the genetic constellation and the coevolution of rotavirus coding genes for the available rotavirus genotypes. Seventy-two full genome sequences of different genetic constellations were analyzed using a genetic algorithm. The results revealed an extensive genome-wide covariance network among the 12 viral proteins. Altogether, the emergence of new genotypes represents a challenge to the outcome and success of vaccination and the coevolutionary analysis of rotavirus proteins may boost efforts to better understand the interaction networks of proteins during viral replication/transcription.

## 1. Introduction

Rotavirus A (RVA) is a double-stranded RNA (dsRNA) virus of the *Reoviridae* family. It is a significant cause of childhood gastroenteritis and accounts for ≈450,000 deaths annually, most occurring in developing countries [1]. RVA causes also a great economic loss to the livestock industry worldwide, and again mostly in the developing countries [2,3]. The triple-layered RVA virion encapsidates an 11-segmented genome that encodes six structural (VP1–VP4, VP6, VP7) and six nonstructural (NSP1–NSP6) proteins [4]. The genotypes of VP7-VP4-VP6-VP1-VP2-VP3-NSP1-NSP2-NSP3-NSP4-NSP5 genes are determined by a classification system proposed by the Rotavirus Classification Working Group (RCWG) [5] and are indicated as Gx-P[x]-Ix-Rx-Cx-Mx-Ax-Nx-Tx-Ex-Hx, where x represents the number of genotypes [6]. From a genetic point of view, the presence of phylogenetically linked constellations of 11 dsRNA segments indicates that the human RVA genes have coevolved to produce protein sets that work optimally together to support virus replication. Studies have revealed numerous distinct genotypes of RVs; some are found throughout the world while others seem to remain regional, and yet others can be seen to emerge, then disappear, only to re-emerge later [7,8]. According to this classification, two virus genogroups were defined: genotype 1 involving human G1P[8], G3P[8], G4P[8], and G9P[8] with invariable internal genes [9,10,11,12] and genotype 2 involving internal genes of human G2P[4]. A third less common genogroup with a limited extent involving G3P[9]. According to the nine internal genes of RVA, genogroup 1–3 are known as Wa-like (I1-R1-C1-M1-A1-N1-T1-E1-H1), DS-1-like (I2-R2-C2-M2-A2-N2-T2-E2-H2), and AU-1-like (G3-P[9]-I3-R3-C3-M3-A3-N3-T3-E3-H3), respectively, with exceptions [13]. Therefore, the evolution of RVA is a complex phenomenon which is driven mainly by accumulation of point mutations due to error-prone genome replication and reassortment. The use of vaccines against the conventional genotypes may constitute additional selection pressure on them and enhance the spread of new genotypes. In addition, it was reported that RVA gene constellations may be influenced by interactions among viral proteins during replication [14].

The aim of the present work was to study the viral fitness with the acquisition of a new gene(s) by analyzing the coevolutionary relationships between RVA proteins and the coevolving residues in the VP1 protein.

Toward this end, advanced computational techniques applied to genome evolution were used to study virus evolution. The resulted genomic data were analyzed and combined with the new available structural and experimental data to investigate the effects of these coevolving residues on the viral replication/transcription.

## 2. Materials and Methods

### 2.1. Sequence Sampling

The available full-length genome sequences of human RVA were retrieved from GenBank (http://www.ncbi.nlm.nih.gov/). We included the available common and uncommon genetic constellations (GC) from different geographical regions at different sampling dates. The associated publications for these sequences were checked and sequence records were retrieved and further verified according to their original publications. We removed sequences with any hypermutation and internal stop codon or ambiguous nucleotide. We excluded very short sequences and sequences from mixed genotype infection. In total, 72 sequences were used in the present study. All strains and isolates were detected during routine surveillance from different countries. The reference strains Wa, DS-1, and AU-1 were included in this study.

### 2.2. Sequence Alignment and Annotation

Each coding gene was preliminarily aligned individually based on the conservation of reading frames by first translating into amino acids using MegaX [15]. Then, we translated the nucleotide coding regions and aligned the resulting amino acid sequences. To obtain the multiple alignments (MSA) of corresponding nucleotide sequences, we mapped the aligned amino acids back to the nucleotide sequences basing on the original nucleotide composition of each gene by DAMBE software version 5.0 [16]. Gaps and ambiguous nucleotides were trimmed from the MSA based on the conservation of reading frames as follows: NSP1 (nucleotides 763–777, 1291–1293, 1435–1437 were trimmed), NSP2 (not trimmed), NSP3 (not trimmed), NSP4 (not trimmed), NSP5 (nucleotides 421–429 were trimmed), NSP6 (not trimmed), VP1 (not trimmed), VP2 (nucleotides 52–54, 70–72, 94–156, 1078–1083 were trimmed), VP3 (not trimmed), VP4 (nucleotides 403–405 and 586–594 were trimmed), VP6 (not trimmed), and VP7 (not trimmed).

The phylogenetic signal of the complete genome dataset was investigated by means of the likelihood mapping using TreePuzzle [17]. A total of 10,000 random quartets (groups of four randomly chosen sequences) were evaluated and, for each quartet, the three possible unrooted trees were reconstructed using the maximum likelihood approach under the selected substitution model. Using the Hasegawa–Kishino–Yano model of substitution [18], the posterior probabilities of each tree were then plotted on a triangular surface: fully resolved trees fall into the corners and the unresolved quartets in the center of the triangle. When more than 30% of the dots fall into the center, the data are considered unreliable for phylogenetic inference.

The retrieved sequences were further analyzed using phylogenetic analysis. Toward this end, firstly we used a pragmatic approach for the detection of recombination, Genetic Algorithm Recombination Detection (GARD) [19], implemented in Spidermonkey [20] through the Datamonkey web-based interface (www.datamonkey.org) [21] and the analysis was validated by *Phi* test using SplitsTree 4 [22]; secondly, we performed the data partitioning schemes as choosing an appropriate partitioning scheme is a central problem for most phylogenetic analyses and it constitutes a crucial step in phylogenetic reconstruction. The MSA was exported in Philip format using Geneious R9 software [23]. We systematically surveyed a number of different data partitioning schemes using PartitionFinder [24]. As the software required a user to predefine partitions and specify in the configuration file, we created an input configuration file that contained a total of 12 partitions, corresponding to the 1st codon, 2nd codon, and 3rd codon of each individual RVA gene. We used the “greedy” algorithm (heuristic search) with branch lengths estimated as “linked” implemented in PartitionFinder to search for the best-fit scheme. All time reversible nucleotide substitution models were tested for each partition. To compare the different partitioning schemes, one needs to choose which model selection approach can be used as a preference. Two criteria can be used to compare multiple models simultaneously, Akaike information criterion (AIC) and Bayesian information criterion (BIC). The models under comparison can be nested or non-nested. There are many papers comparing the merits of the different metrics [25,26]. The optimal model-choice strategy is to calculate the BIC score based on the empirical log-likelihood and choose the model with the lowest score [27]. The obtained substitution model was further evaluated and simplified by generating NJ tree for each RVA gene using PAUP * v.4 software [28]; finally, we carried out phylogenetic analysis using maximum likelihood (ML) method and the best tree was picked up according to their ML score using Iqtree v1.6.12 software [29]. The branch support was carried out using four methods: approximate Bayes test [30], ultrafast bootstrap (UFBoot) [31], Shimodaira–Hasegawa-like approximate likelihood ratio test (SH-like aLRT) [32], and local bootstrap probabilities method (LBP) [33].

Finally, 72 full genome sequences were selected and annotated using the Sequence Name Annotation-based Designer (SNAD) [34]. The annotations of sequences include strain or isolate name, country, date of collection, and genetic constellation. The annotations were adjusted later manually for expected errors and the accession numbers of sequences were replaced by their appropriate annotations using Javascript (Table S1).

### 2.3. Coevolving Protein Residues and Calculation of Codon *dN/dS* Values

We wondered whether the amino acid changes detected in one viral protein correlated with changes in the same (inter-coevolving sites) or in different viral proteins (intra-coevolving sites), as such intermolecular covariation is indicative of protein coadaptation [35,36].

An individual alignment was created for each of the 12 RVA proteins (VP1–4, VP6,7, NSP1–6) to be used for intra-coevolutionary analysis. For the inter-coevolutionary analyses; the amino acid sequence alignments of the RVA proteins were concatenated with a Bioperl script, using all possible protein combinations (*n* = 66).

To identify significant intermolecular connections, we applied the Bayesian Graphical Models (BGM) method implemented in Spidermonkey [37] through the Datamonkey web-based interface. A BGM is a compact representation of the joint probability distribution in which each node represents a distinct random variable. An edge originating from two nodes postulates a conditional dependence between the corresponding sites. The given algorithm uses the Markov chain Monte Carlo (MCMC) algorithm [38] to infer the configuration of edges in the graph that best explains the data. The dN/dS values for each codon were determined and the codon-aligned nucleotide sequences of the complete open reading frames for all genes using SNAP v.1.1.1 [39,40,41,42].

In order to combine the coevolving sites with the protein MSA variability, the evolutionary conservation at each site in multiple sequence alignment was calculated by sum-of-pairs measure with an independent count weighting scheme implemented in the AL2CO program [43]. Blosum62 was used as a scoring matrix and the window size used for averaging conservation was set to 3 for motif analysis. The obtained results were plotted using the “R” environment [44].

## 3. Results

### 3.1. Recombination and the Choice of the Model of Evolution

For each alignment, likelihood mapping analysis showed very low level of phylogenetic noise, indicating sufficient information for reliable phylogeny inference (Figure S1).

The substitutional process underlying the evolution of RVA genes seems to be similar, following the General Time-Reversible (GTR) model with invariant sites (I) and with Gamma-distributed among site rate variation (G) based upon the results of BIC scores as implemented in PartitionFinder. The GTR + I + G, model also had the highest likelihood score for each gene. The *Phi* test and GARD did not show statistically significant evidence of recombination. The ML phylogenetic tree is highly resolved, with most of tree branches supported by all four bootstrap methods (Figure S2).

### 3.2. Intermolecular Interactions of RVA Proteins

According to AIC and the natural log of the likelihood, the evolutionary model JJT (Jones, Taylor, Thornton) + F was the best fit for the present data and, therefore, used to perform BGM analysis. We, conservatively, considered only sites with posterior probability ≥0.9, although we cannot exclude that the number of protein interactions is, in fact, larger. 

The results of intra-coevolutionary analysis identified 46 sites involved in 31 interactions in NSP1, four sites for two interactions in NSP2, two sites for one interaction in NSP3, five sites for three interactions in NSP4, two sites for one interaction in NSP5, two sites for one interaction in NSP6, 14 sites for eight interactions in VP1, eight sites for four interactions in VP2, 33 sites for 20 interactions in VP3, 20 sites for 13 interactions in VP4, three sites for two interactions in VP6, and 17 sites for 10 interactions in VP7 (Figures S3 and S4). The results show that the majority of the covarying amino acid positions are encoded by codons with dN/dS ratios of >1, suggesting that the selection is driving the fixation of specific protein residues. However, no sites under selection were shown for NSP5. For VP1, 78.5% of covarying amino acid positions were associated with codons that showed dN/dS ratios of >1; VP2—75%; VP3—79%; VP4—90%; VP6—67%; VP7—70%; NSP1—98%; NSP2—75%; NSP3—100%; NSP4—60%; NSP6—100% (Table 1). Some of the intra-coevolving sites are involved in the intermolecular interactions as well, mainly sites mapped to VP1-4, NSP1, and NSP4. The 14 intra-coevolving sites of VP1 areas were distributed as follows: two interactions within the N-terminal domain, one interaction within polymerase domain, one interaction between N-terminal domain and polymerase domain, two interactions between polymerase domain and C-terminal domain, and two interactions between N-terminal and C-terminal domains.

**Figure 1:**
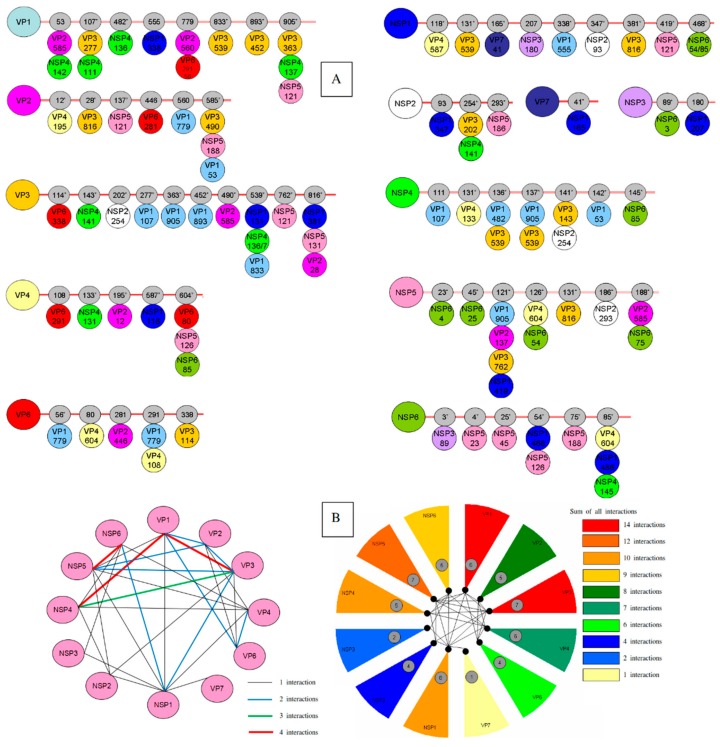
Coevolutionary analysis of Rotavirus A (RVA) proteins. (**A**) Linear representation of intermolecular connection. Each viral protein is represented by a circle and residue position with a defined color. Grey circles on the horizontal lines represent the positions of coevolving residues. *dN*/*dS* ratios of >1 is represented by stars. (**B**) Intermolecular covariation network. Each viral protein is represented by a pink circle. Lines connecting the circles indicate that the two proteins showed intermolecular covarying amino acid positions with posterior probability ≥0.9.

The interdependent quantification between all concatenated amino acid sequence alignments revealed an extensive covariance network involving amino acid positions in all 12 RVA proteins. VP1 interacts with six proteins through 13 interactions. The covariation analysis revealed that the number of intermolecular connections was highest between VP1, VP3, and NSP4; yet NSP5 and its internal ORF, NSP6 (Figure 1A,B). The highest intermolecular interactions were shown for NSP1 (eight interactions) with VP1, VP3/VP4, VP7, NSP2/NSP3, and NSP5/6. Besides, NSP5 showed intermolecular interactions with two structural proteins, VP3 and VP4; NSP6 showed interactions with NSP3 and VP4; VP3 showed interactions with NSP2 and VP6; VP1 showed interaction with VP6. Additionally, an unexpected intermolecular interaction was detected between VP2 and VP4. 

Interestingly, some proteins showed coevolving sites with more than one protein at the same position with most of these sites under positive selection (Figure 1A), mapping to VP1 (positions 53, 107, 779, and 904), VP2 (position 585), VP3 (positions 539 and 816), VP4 (position 604), VP6 (position 291), NSP2 (position 254), NSP4 (positions 136, 137, and 141), and NSP5 (positions 121, 126, and 188).

For VP1, 62.5% of intermolecular sites were associated with codons that showed dN/dS ratios of >1; VP2—67%; VP3—100%; VP4—80%; VP6—20%; VP7—100%; NSP1—89%; NSP2—67%; NSP3—50%; NSP4—86%; NSP5—100%, NSP6—100%.

### 3.3. Mapping of the Interacting Sites

The coevolving sites were mapped to the primary structure of RVA proteins. In addition, the inter-coevolving sites were mapped on the available tertiary structures of proteins. These 3D structures were available for NSP2, VP1, VP2, VP4, VP6, and VP7. Most of the interacting sites are surface exposed with some exceptions. 

NSP1. The overall coevolving sites within NSP1 protein are mapped to the N- and C-terminal of the protein. However, the majority of sites are within the region between the RNA binding domain (RNA-BD), the interferon regulatory factor 3 binding domain (IRF3-BD), and C-terminal region downstream IRF3-BD (Figure S5G). Two out of the four coevolving sites within RNA-BD are mapping in the zing binding ring domain (RING) (sites 55 and 70). For simplicity we named the region between RNA-BD and IRF3-BD as region A; the C-terminal domain downstream IRF3-BD as region B.

The highest numbers of interacting sites within NSP1 are between the region A and region B (*n* = 10); seven interactions within region B; five interactions within region A; three interactions between region A and IRF3-BD; two interactions between IRF3-BD and region B; two interactions between RNA-BD and region A; one interaction within RNA-BD; one interaction between RNA-BD and region B. The inter-coevolving sites are mapped to region A (*n* = 4), IRF3-BD (*n* = 2), and region B (*n* = 3). The coevolving sites in region A interact with VP3-4, VP7, and NSP3; coevolving sites of region B interact with VP3 and NSP5-6; coevolving sites of IRF3-BD interact with VP1 and NSP2. All the intermolecular sites were encoded by codons with dN/dS ratios of >1, except aa 207. Due to the lack of a 3D structure of NSP1, we could not map these intermolecular sites to further study their localization within the 3D protein structure.

NSP2. Most of the coevolving sites are mapped to the C-terminal of NSP2. The only coevolving site mapping to the N-terminal domain of the protein (aa 93) showed interaction with residue 347 of NSP1 (Figure S5H). Residue 191 is mapped to the β-strand 7 (aa 186 to 191), which constitutes with β-strand 9 (aa 226 to 230) and the internal loop (aa 221 to 226) the base of the deep cleft in NSP2 protein [45]. In addition, residue 191 is mapped closed to the active site for NTP hydrolysis [46,47,48] Three coevolving sites (aa 245, aa 254, and aa 256) are mapped to the C-terminal internal loop extended from aa 245 to 260 which constitutes the C-terminal side of the cleft [45] and antibody-binding epitope [49]. Residue 254 is mapped to one-side of the cleft and coevolves with aa 141 of NSP4 and aa 202 of VP3. Residue 293 is mapped to the C-terminal of the protein and coevolves with aa 186 of NSP5. Three surface-exposed inter-coevolving sites (aa 93, aa 254, and aa 393) are mapped to loop structures (Figure S6). The intermolecular sites at aa 254 and aa 293 were encoded by codons with dN/dS ratios of >1 whereas site 93 was not. All coevolving sites lie on the least conserved regions of RVA proteins.

NSP3. It undergoes two intra-coevolving sites (aa 186 and aa 309). According to its structural data [50], these sites are mapped to the dimerization domain and Eukaryotic translation initiation domain (eIF4G-BD) (Figure S5I). None of these sites are involved in intermolecular interactions. Sites 89 and 180, however, are involved in interactions with NSP6 and NSP1, respectively (Figure 1) and the only site at aa 180 was encoded by codon with dN/dS ratios of >1. All coevolving sites lie on the least conserved regions of RVA proteins (Figure S7).

NSP4. The cytoplasmic tail of NSP4, extended from aa 45 to aa 175, exhibits all of the known important biological functions [51]. According to the available gene organization of NSP4 [51,52], all the coevolving sites are mapped to the C-terminal of the protein which attains a cytoplasmic orientation: seven sites in the VP4 binding domain (VP4BR) including interspecies variable domain (ISVD) (four sites); one site mapped to heptad repeat region (HRR) upstream VP4BR region; two sites in double-layered particle binding domain (DLPBR) downstream VP4BR region (Figure S5I). Seven out of these 10 sites are involved in intermolecular interactions with RVA proteins and all are mapped to HRR and ISVD domains. Most of these interactions are with VP1/VP3 proteins (seven out of 10) followed by VP4, NSP6, and NSP2 with one interaction for each. The amino acid 131 of NSP4, mapping to VP4BD, interacts with VP4 whereas aa 141 and aa 145 interacts with NSP2 and NSP6, respectively (Figure 1 and Figure S5J). Moreover, the site at aa 131 is mapped in the calcium-binding domain extended from aa 114 to aa 135, known to play an important role in stabilizing the tetramer structure of NSP4 and its engagement as an enterotoxin [52]. Due to the lack of the complete 3D structure of NSP4, we could map only two sites (amino acid residues 111 and 131) (Figure S8). Most of these sites lie on the least conserved regions of RVA proteins. The intermolecular site 131 was encoded by codons with dN/dS ratios of >1 whereas site 111 was not.

NSP5/6. NSP5 undergoes nine coevolving sites (Table 1 and Figure 1) mapping mainly to N- and C-terminal regions of the protein (Figure S5K). Seven out of the nine sites are involved in intermolecular interactions with VP1-4, NSP1-2, and NSP6. Interestingly, most of these intermolecular interactions of NSP5 are mapped to the C-terminal of RVA proteins (Figure 1). Four out of the seven interacting sites are mapped to the two oligomerization regions of NSP5 mapping to aa 103–146 and aa 188–198 [53].

The remaining two intra-coevolving sites are mapped to the oligomerization domain extended from aa 103 to aa 146 (site 112) and Fe-S cluster reported to modulate the interaction of NSP5 to RNA [54] (site 177). All intermolecular sites of NSP5 were encoded by codons with dN/dS ratios of >1 and lie on the least conserved regions of RVA proteins (Figure S7).

VP1. The 17 coevolving sites are mapped to N- and C-terminal of the protein (Figure S5A). Fourteen out of the 17 coevolving sites are intra-coevolving sites responsible for eight interactions: two interactions within N-terminal domain, one interaction within polymerase domain, one interaction between N-terminal domain and polymerase domain, two interactions between polymerase domain and C-terminal domain, and two interactions between N-terminal and C-terminal domains [55,56]. The remaining three coevolving sites (aa 779, aa 893, and aa 905), with five intra-coevolving sites (aa 53, aa 107, aa 482, aa 555, and aa 833), constitute the intermolecular interactions with VP2/3, VP6, NSP1, and NSP4/5 (Figure 1). The highest number of interactions is shown with the capping enzyme, VP3 (four interactions). All these eight intermolecular sites are surface exposed (Figure S9). Six out of the coevolving sites are mapped to α-helix domains (residues 107, 482, 555, 779, 833, and 905) whereas the remaining two sites are mapped to loop regions (residues 53 and 893). Amino acid residues 833 and 779 lie in the dsRNA/(−)RNA exit tunnel whereas aa 107 lies in the nucleoside triphosphate (NTP) entry tunnel (Figure S9). Amino acid residues 482 and 555 lie in the (+)RNA exit tunnel. The intermolecular amino acid residues 107, 482, 833, 893, and 905 were encoded by codons with dN/dS ratios of >1 whereas amino acid residues 53, 555, and 779 were not. All of them lie on the least conserved regions of RVA proteins.

VP2. The protein contains two major domains: the N-terminal domain (aa 1–100) and a principal domain (aa 101–880) for polymerase activation. The N-terminal domain formed by a five-fold hub (aa 1–80) and a linker (aa 81–100) [57,58]. The 12 coevolving sites of VP2 are mapped to both domains with five coevolving sites on the N-terminal domain (amino acid residues 12, 28, 39, 40, and 67) and seven coevolving sites on the principal domain (amino acid residues 128, 137, 214, 229, 446, 560, and 585) (Figure S5B). All the coevolving sites on the N-terminal domain are mapped to the five-fold hub subdomain. 

All eight intra-coevolving sites (Table 1) are responsible for four interactions between the N-terminal and the principal domain. Among them, two sites (amino acid residues 12 and 446) and four other sites (amino acid residues 28, 137, 560, and 585) are involved in intermolecular interactions with five RVA proteins (VP1, VP3, VP4, VP6, and NSP5) (Figure 1). Among these six coevolving sites, four are mapped to the 3D structure and it was not possible to map the remaining two sites lack of this region on the available partial structure. Two out of the four sites are mapped to the inner interface of the protein surface (amino acid residues 137 and 585) (Figure S10). A cut-away view of the VP2 dimer was used to show the hidden sites (amino acid residues 446 and 560). Amino acid residues 446, 560, and 585 are mapped to the two-fold axis of VP2 dimer and five-fold axis of VP2 decamer, a region known for its interaction with the replication complex formed by VP1-VP3 whereas amino acid residue 137 is mapped to the far extremity of the protein (Figure S10). The intermolecular sites (amino acid residues 12, 28, 137, and 585) were surface exposed and encoded by codons with dN/dS ratios of >1 whereas the hidden amino acid residues 446 and 560 were not. All sites lie on the least conserved regions of RVA proteins.

VP3. The protein undergoes the second highest number of coevolving sites, after NSP1. It is constituted by the N-terminal domain and four main enzymatic domains: guanine-N7-methyltransferase (N7-MTase), ribose-2′-*O*-methyltransferase (2′-*O*-MTase), guanylyltransferase/RNA 5′-triphosphatase (Gtase/RTPase), and phosphodiesterase (PDE) [59]. The 33 intra-coevolving sites are mapped to all protein domains with eight sites on the N-terminal domain, 11 sites on the M7-MTase, six sites on the 2′-*O*-MTase, one site on the Gtase/RTPase, and seven sites on the PDE (Figure S5C). Six out of the 33 intra-coevolving sites and four other sites are involved in intermolecular interactions with RVA proteins (Figure 1). Most of the inter-coevolving sites of VP3 are shown with VP1 (four interactions). All intermolecular sites of VP3 were encoded by codons with dN/dS ratios of >1 and lie on the least conserved regions of RVA proteins (Figure S7).

VP4. According to the available structural data [60,61,62], the 20 intra-coevolving sites are mapped to the three main domains: lectin domain (aa 20–231)—eight sites; β-barrel (aa 248–510)—six sites; C-terminal domain (aa 510–776)—six sites (Figure S5D). These sites are responsible for four interactions within the lectin domain, one interaction within the β-barrel domain, two interactions between the lectin and β-barrel domains, and four interactions between the β-barrel and the C-terminal domains. Two out of these intra-coevolving sites and three other sites (amino acid residues 108, 195, and 587) are involved in intermolecular interactions with VP2, VP6, NSP1, and NSP4/5/6 (Table 1 and Figure 1). Four out of the five intermolecular sites lie on the surface of the protein, formed by loops; whereas aa 108, located in the β-strand, is mapped deep inside VP4 protein structure (Figure S11). Amino acid residues 108, 133, and 195 are located in the globular domain VP8 formed after the cleavage of VP4 following trypsin activation. The second generated protein after cleavage, VP5, undergoes the remaining two residues (amino acid residues 587 and 604). These residues lie within the VP5 foot. The four surface exposed residues, but not aa 108, were encoded by codons with dN/dS ratios of >1. All sites lie on the least conserved regions of RVA proteins (Figure S11).

VP6. The three intra-molecular sites (amino acid residues 199 and 252) are mapped to the H domain of VP6 protein; whereas aa 130 lies on the B subdomain extended from aa 1 to aa 150 (Figure S5E) [63]. These sites are mapped to the trimerization domain (aa 105–328) [64]. They are not involved in intermolecular interactions with RVA proteins. Instead, surface exposed sites 50, 80, 281, 291, and 338 are involved in intermolecular interactions with four structural proteins VP1/2/3/4 (Figure 1 and Figure S12). Among them, two residues are mapped to the region of interaction with the outer layer (amino acid residues 281 and 291) (Figure S5E). Only the intermolecular site 53 was encoded by a codon with dN/dS ratios of >1 and all of them lie on the least conserved regions of RVA proteins (Figure S12).

VP7. The 17 intra-interacting sites (Table 1) lie on the N-terminal domain of the protein (eight sites), mainly at variables sites VR1, VR2, VR3, and VR4. Six sites are mapped to the Rossman-fold domain and three sites to the B-jelly domain of the protein (Figure S5F) [65]. Most of these intra-molecular sites are localized in the variable regions [66] and in the serotype-specific antigenic sites A, B, and C [67]. However, none of these sites are involved in intermolecular interactions. The lack of a complete 3D structure of VP7 makes impossible to localize the coevolving site 41. However, according to the available structure, the site has to be mapped on the loop structure of the protein within the region exposed to DLP (Figure S13). The intermolecular site 41 was encoded by codons with dN/dS ratios of >1. Most of the coevolving sites lie on the least conserved regions of RVA proteins.

In order to verify whether the intermolecular sites on VP1, VP2, VP4, and VP6 could be a reflection of physical interactions, we mapped these sites on the 3D structures of these proteins (Figures S14 and S15). Interestingly, the intermolecular interactions are found at very far spatial proximity of proteins.

## 4. Discussion

Our analyses of the coevolutionary dynamics within RVA proteins, as well as between them, uncover a complex network of evolutionary dependencies among amino acid sites. These dependencies often involve sets of sites with known functional relevance but also comprise other sites with unknown importance. In addition, the lack of structural data for some RVA proteins hampers our effort to better understand the position significance of these coevolving sites.

Using coevolution analysis of RVA proteins, we expected that residues mapping in proximity with each other in a native structure, or coming into contact upon folding, would not evolve independently from each other, due to the need to maintain amino acid interactions important for protein stability and foldability. Indeed, amino acid residues involved in intermolecular interactions among VP1, VP2, VP4, and VP6 mapped on proteins’ 3D structures (using available crystallographic data) were spatially far. It was reported that the interactions of RVA proteins during the replication process may induce conformational changes of proteins generating new protein isoforms that may contribute to the formation of several replication intermediates varying in both shape and sizes [68]. Thus, the interacting sites between RVA proteins cannot be considered stable and might change in the course of the replication process. In addition, it is possible that these viral proteins do not actually interact but although they can independently, yet synergistically, affect viral fitness [35].

The largest number of protein interactions were shown for the nonstructural protein NSP1 (eight viral proteins: VP1, VP3, VP4, VP7, NSP2, NSP3, NSP5, and NSP6) (Figure 1). Although it was reported that NSP1 is not required for RVA replication, NSP1 does interact in vivo with the four nonstructural proteins (NSP2, NSP3, NSP5, and NSP6) [69,70,71], and in silico study showed a high number of interactions of NSP1 with practically all the components of the replication [72]. Altogether, these findings enhance further efforts to better understand the role of NSP1 during viral replication. Due to the lack of structural studies on NSP1, only a brief description of its function is included in this section. Some uncapped or incompletely capped (+)RNA molecules, upon their release from double-layered particles (DLP), are not absolutely secure from degradation when exposed to the cytoplasmic content of the cell during the early stages of infection by activating interferon (IFN) response [73,74]. Therefore, in order to protect viral RNAs, NSP1 functions as an antagonist of the host IFN response to protect the virus from the innate immune response by binding to more than one interferon regulatory factor (IRF) [75]. By looking to these interactions, shown in the present work, we can find that NSP1 interacts with VP1–VP3 forming a protein complex for transcription; with VP4–VP7 necessary for the last stage of particle coating and triple-layered particle (TLP) formation; with NSP2–NSP5/6 necessary for viroplasm formation; yet with NSP3. In addition, it was reported that RVA protein VP7 was retained by glutathione S-transferase (GST) pull-down assay combined with IRF type 3 (GST-IRF3). It is possible that VP7 is bound by NSP1 complexed to IRF3, rather than IRF3 alone [76]. Knowing that the secondary structures of RVA RNAs show cis-acting signals formed by the 5′- and 3′- end of the untranslated regions with extend to coding regions and constitute a panhandle structure that promotes the synthesis of dsRNA [77], it is possible that due to their affinity to RNA, NSP1, and VP1-VP3 may interact and the viral RNAs may be the intermediates in these interactions. It is possible that the spread of NSP1 protein in the cytoplasm [78] leads it to interact with most of the viral replication components.

The nonstructural protein NSP5 showed interactions with seven viral proteins (VP1, VP2, VP3, VP4, NSP1, NSP2, and NSP6). This protein was shown to interact with NSP1 [71], VP2 [79], necessary for its phosphorylation [80], with NSP6 [81], and VP1 [82]. In addition, the present analysis showed intermolecular interactions between NSP5 and another component of viroplasm, VP3, suggesting that NSP5 may play an important role in protein recruitment and assembly, rather than viroplasm formation and RNA interaction. Interestingly, NSP5 showed a coevolving site (aa 126) with VP4 (Figure 1), yet shared by NSP6. Again, the lack of tertiary structures of NSP5 and NSP6 hampers our efforts to better study these interactions and to determine whether this interaction is via NSP6 protein. The latter, not expressed by all viral strains, showed coevolving sites with nonstructural protein NSP4. During early infection, NSP4 protein exerts a proapoptotic effect on host cells by interacting with mitochondrial proteins adenine nucleotide translocator and voltage-dependent anion channel, resulting in dissipation of mitochondrial potential, the release of cytochrome c from mitochondria, and caspase activation [83]. The function of NSP4 needs to be regulated at this stage to keep virus replication within infected cells. Therefore, this apoptosis activation by NSP4 is inhibited by the activation of cell survival pathways (PI3K/AKT) induced by NSP1, as an antagonist to NSP4 protein. The localization of NSP6 with the cell mitochondria and its possible interaction with mitochondrial proteins [84] may support our finding that NSP6 may interact with NSP4. However, the function of NSP6 in viral replication for some viral strains is still unknown and further analysis needs to be undertaken in order to elucidate its function as well as its strain-dependent expression.

Interestingly, the present study also found coevolving residues between VP4 and VP2, in agreement with a previous study [72], although no data about their interactions during viral replication is currently known.

Most of the coevolving sites in VP1 interacted with VP3 and NSP4, followed by VP2. Although coevolving residues between VP1 and VP3/VP2 are expected, the finding that VP1 residues coevolve also with NSP4 was unexpected, since it is known that NSP4 functions as an intracellular receptor in the endoplasmic reticulum membrane (ER) [85] that may play an important role in virion assembly at the late stages of viral replication according to different hypotheses [86,87,88]. However, it was reported recently that NSP4 can accumulate in proximity of viral replication components, mainly NSP2, NSP5, VP1, VP2, and VP6 [89], suggesting that it also plays an important role as a regulator of viral particle assembly. In addition, intra-co-evolving residues within VP1 were located to the N- and C-terminal of the protein, as supported by a recent report showing that N- and C- terminal domains of VP1 regulate its function [90].

## 5. Conclusions

Several studies on the interactions between RVA proteins have improved our understanding of the mechanism of RVA genome replication, yet our knowledge of the details of these interactions remains limited. Our results further strengthen the hypothesis of a complex network of interactions implicating all RVA proteins.

We studied the molecular interactions within and among RVA proteins using full genomes from different RVA genotypes detected in different geographic regions during a long time period. Our analyses of the coevolutionary dynamics within RVA proteins, as well as between them, uncover a complex network of evolutionary dependencies among amino acid sites. These dependencies often involve sets of sites with known functional relevance, but also comprise other sites with unknown importance due to lack of structural and functional data for some RVA proteins. Our findings provide support for experimental investigations to characterize further the interactions between RVA proteins during RNA replication and virion assembly.

## Figures and Tables

**Table 1:** Coevolutionary analysis of rotavirus proteins performed by Bayesian genetic method.

Proteins	Residues (Intra-Coevolving Sites)	Residues (Inter-Coevolving Sites)
VP1	51*-**53**-107*-120-156*-293*-294*-357*-482*-**555**-657*-833*- 891*-1044*	**53**-107*-482*-**555**-779-833*-893*-905*
VP2	12*-39*-40*-67*-128*-214*-229-**446**	12*-28*-137*-**446**-560-585*
VP3	54*-88-89-109*-114*-115*-116-143*-202*-203*-204*-205*-245*-266*-277*-336*-363*-373-405*-430*-437*-438-455*-478*-539*-625*-706*-707*-716-749*-751*-767*-798	114*-143*-202*-277*-363*-452*-490*-539*-762*-816*
VP4	106*-121*-131*-133*-135*-145*-150*-192-254*-280-283*-305*-337*-444*-586*-604*-630*-674*-713*-750*	108-133*-195*-587*-604*
VP6	130*-199*-252	56*-80-281-291-338
VP7	32-37*-50-57-68*-72*-73*-74*-94*-130-139*-149*-193-212*-237*-291*-303*	41*
NSP1	10*-19*-55*-70*-93*-96*-108*-121*-163*-166*-180*-219*-223*-224*-225*-230*-253*-266*-268*-277*-293*-297*-312*-314*-326*-347*-357*-371*-372*-373*-381*-383*-388*-391*-402*-408*-419*-422*-435-436*-438*-440*-441*-459*-463*-476*	118*-131*-165*-207-338*-347*-381*-419*-468*
NSP2	191-245*-256*-314*	93-254*-293*
NSP3	186*-309*	89*-180
NSP4	141*-145*-148-169*-174	111-131*-136*-137*-141*-142*-145*
NSP5	112-177	23*-45*-121*-126*-131*-186*-188*
NSP6	75*-88*	3*-4-25-54*-75*-85*

Sites showed both inter- and intra-coevolving sites are shown in bold; positions of intra-coevolutionary sites are based on those of strain Wa (JX406747-JX406757). Sites with dN/dS ratios of >1 are shown by stars (*).

## Supplementary Materials

The following are available online at https://www.mdpi.com/2073-4425/11/1/28/s1, Figure S1: Likelihood mapping of the VP1 sequences, Figure S2: ML phylogenetic tree for sequence data set, Figure S3: Matrix of protein pairs connections, Figure S4: Genetic Algorithm Recombination Detection (GARD) for intra-coevolving residues, Figure S5: The position of coevolving sites on the primary structure of proteins. The position of functional and structural domains are shown by different colors and annotated as reported in the literature, Figure S6: The atomic structure of NSP2 monomer, Figure S7: Conservation plot analysis at each site in multiple sequence alignment using weighted sum-of-pairs measure, Figure S8: The partial atomic structure of NSP4, Figure S9: The 3D structure of VP1 protein, Figure S10: The atomic structure of VP2 monomer, Figure S11: The atomic structure of VP4, Figure S12: The atomic structure of VP6 monomer, Figure S13: The atomic structure of partial VP7 monomer, Figure S14: The atomic structure of protein complex formed by VP4, VP6, and VP2, Figure S15: The atomic structure of protein complex formed by VP6, VP2, and VP1; Table S1: Accession numbers, internal gene type, and genetic constellations of all sequences included in the study.
