# Supplementary material for: Coding-Gene Coevolution Analysis of Rotavirus Proteins: A Bioinformatics and Statistical Approach
Source: Genes (Basel). 2019 Dec 24;11(1):28. doi: 10.3390/genes11010028 (PMC7016848; doi:10.3390/genes11010028)

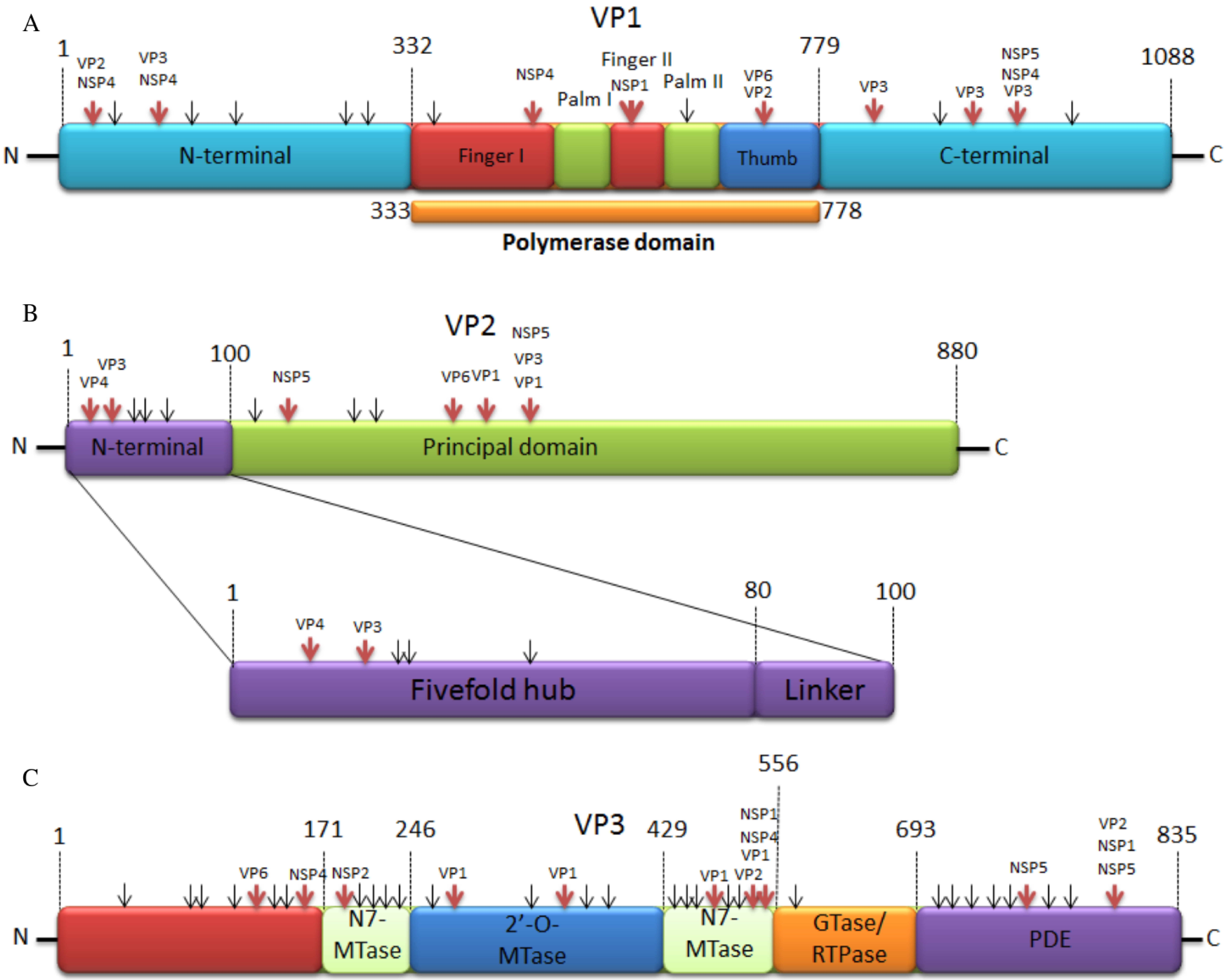

N7-MTase: guanine-N7-methyltransferase; **2′-O-MTase**: ribose-2′-O-methyltransferase; **GTase/RTPase**: guanylyltransferase/RNA 5′-triphosphatase; **PDE**: phosphodiesterase.

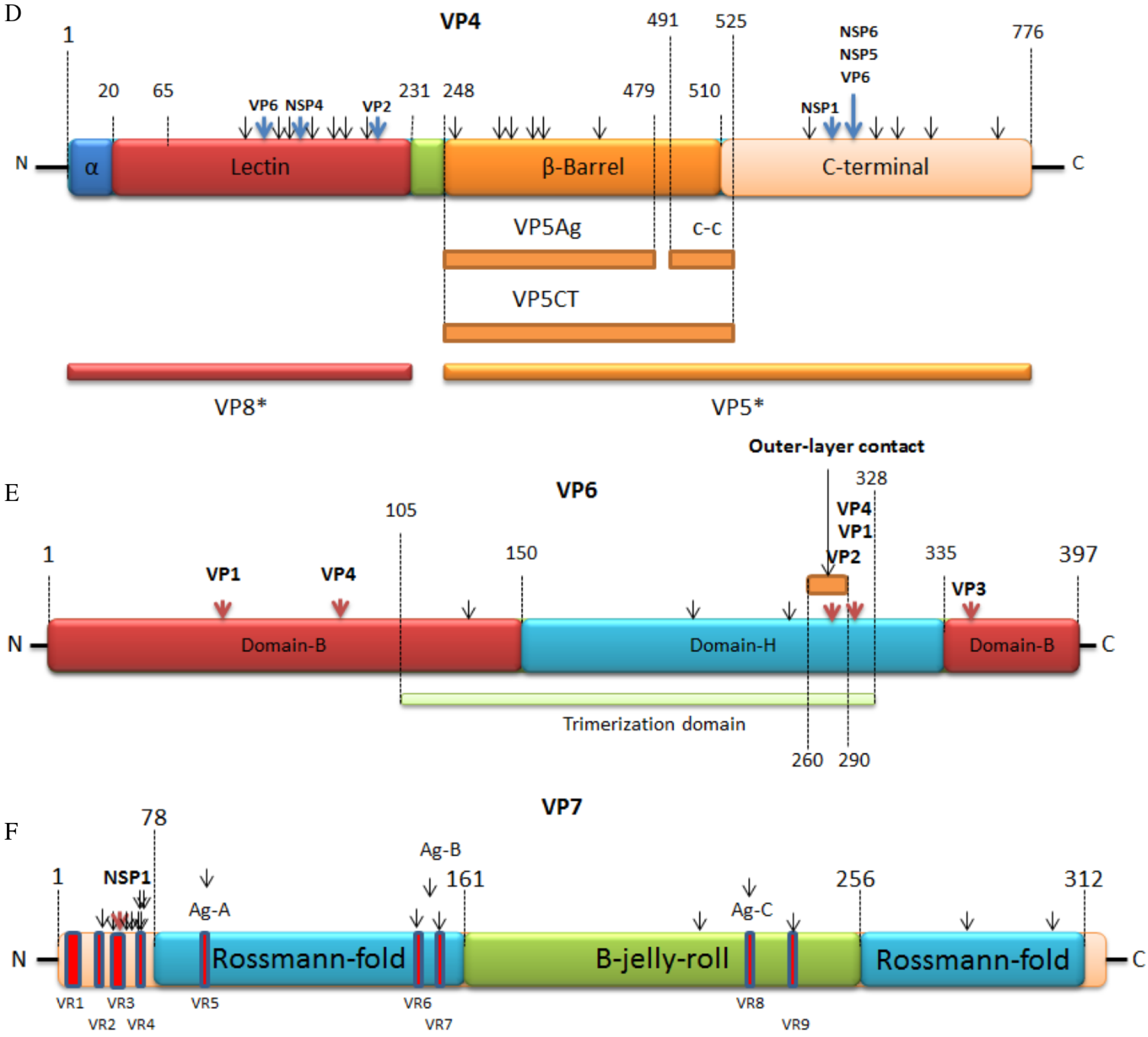

VR: Variable regions. **VR1**: 9-20; **VR2**: 25-32; **VR3**: 37-53; **VR4**: 65-76; **VR5**: 87-100; **VR6**: 119-132; **VR7**: 141-150; **VR8**: 208-224; **VR9**: 235-242. **Ag**: Serotype-specific antigenic sites. **Ag-A**: aa 87-96; **Ag-B**: 145-150; **Ag-C**: 211-223.

G

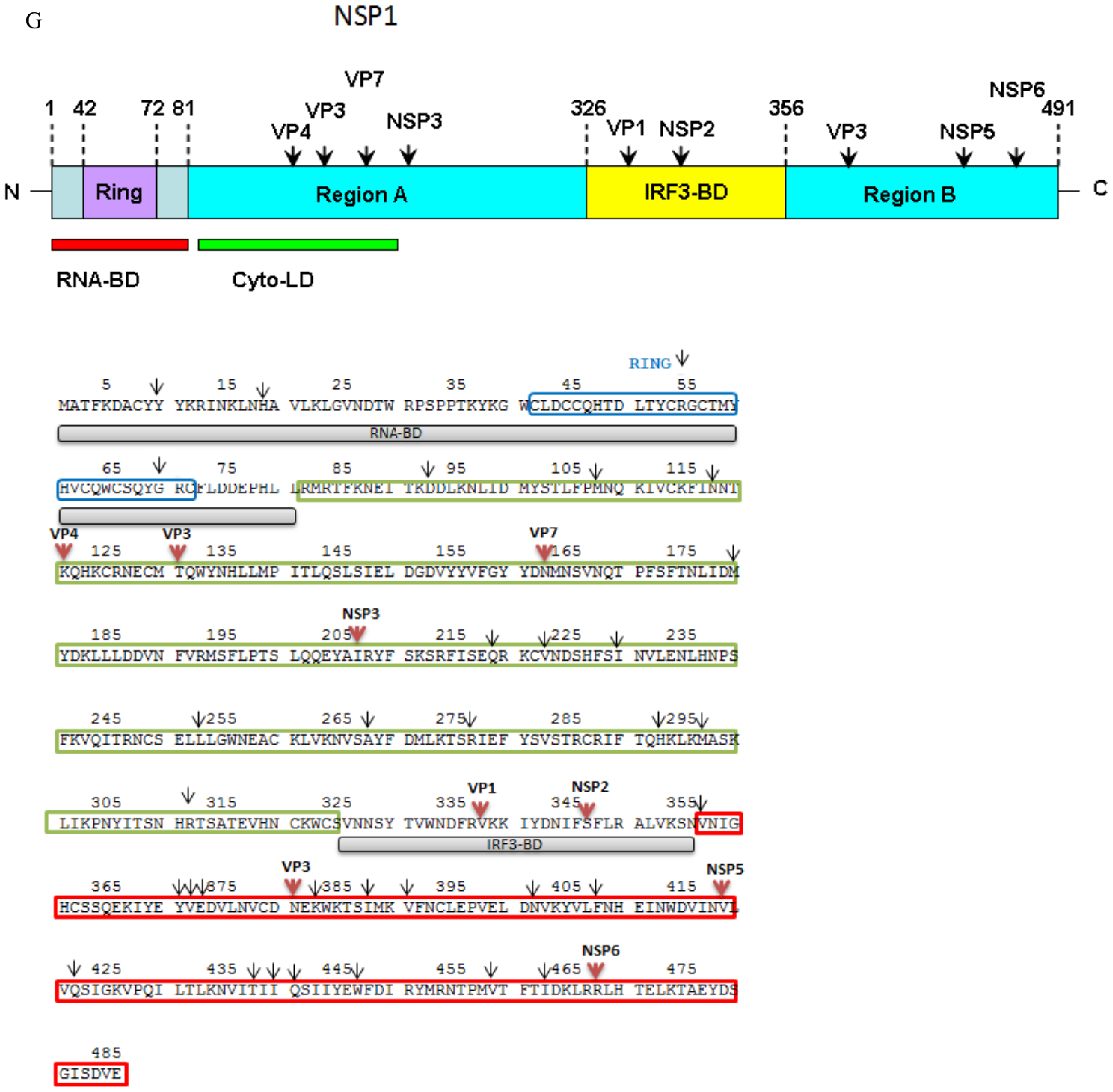
NSP1
VP7
1 42 72 81
VP4 VP3 NSP3
326
VP1 NSP2
356
VP3 NSP5
NSP6
491
N
Ring
Region A
IRF3-BD
Region B
C
RNA-BD
Cyto-LD
RING
MATFKDACYY YKRINKLNHA VLKLGVNDTW RPSPPTKYKG WCLDCCQHTD LTYCRGCTMY
RNA-BD
HVCQWCSQYG RCFLDDEPHL LRMRTFKNET TKDDLKNLTD MYSTLFPMNQ KIVCKFINNT
VP4 VP3 VP7
KQHKCRNECM TQWYNHLLMP ITLQSLSIEL DGDVYYVFGY YDNMNSVNQT PFSFTNLIDM
NSP3
YDKLLLDDVN FVRMSFLPTS LQQEYAIRYF SKSRFISEQR KCVNDSHFSI NVLENLHNPS
FKVQITRNCS ELLLGWNEAC KLVKNVSAYF DMLKTSRIEF YSVSTRCRIF TQHKLKMASK
VP1 NSP2
LIKPNYITSN HRTSATEVHN CKWCSVNNSY TVWNDFRVKK IYDNIFSFLR ALVKSNVNIG
IRF3-BD
VP3 NSP5
HCSSQEKIYE YVEDVLNVCD NEKWKTSIMK VFNCLEPVEL DNVKYVLFNH EINWDVINVL
NSP6
VQSIGKVPQI LTLKNVITII QSIIYEWFDI RYMRNTPMVT FTIDKLRRLH TELKTAEYDS
GISDVE

H

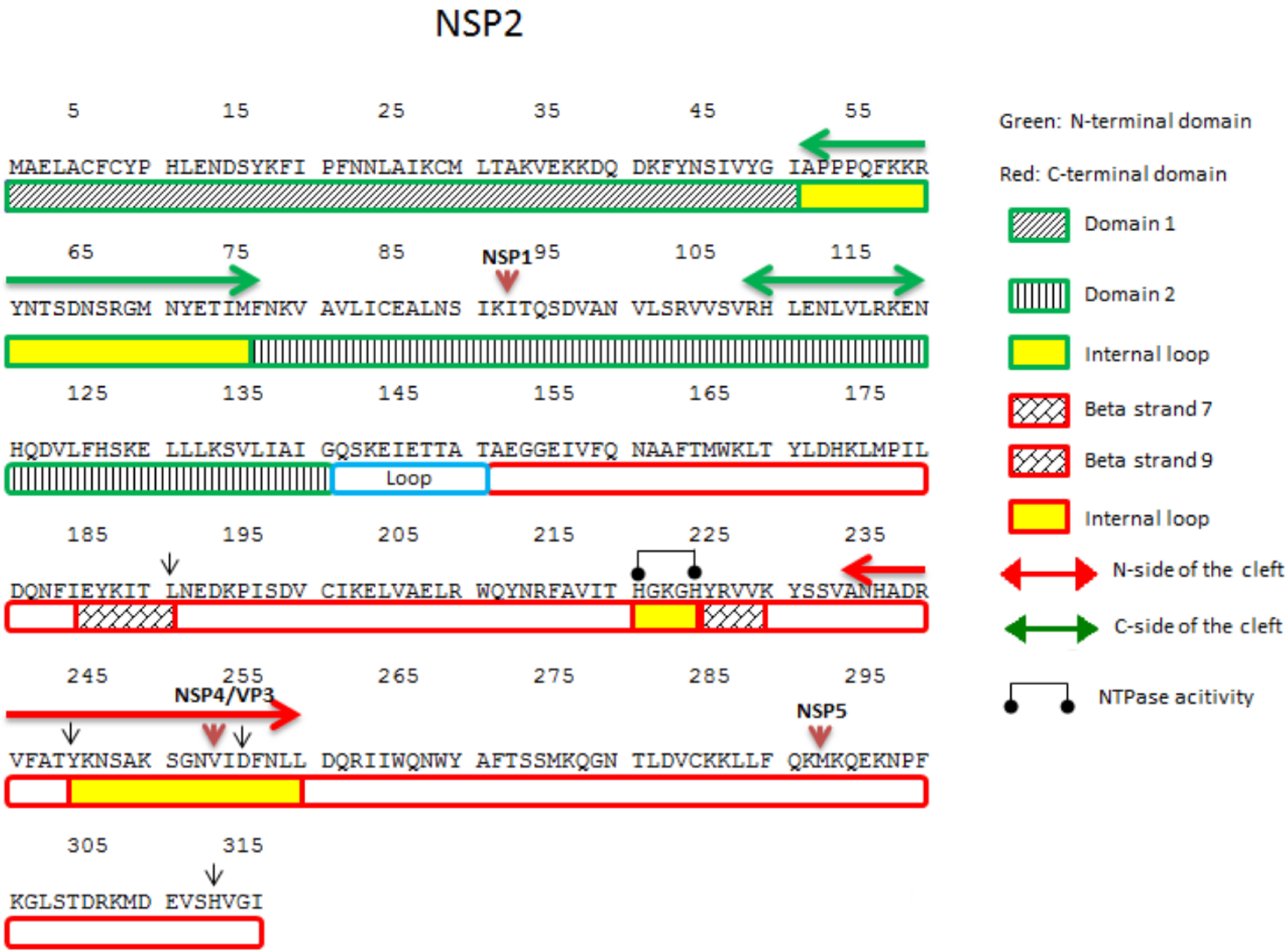
NSP2
5 15 25 35 45 55
MAELACFCYP HLENDSYKFI PFNNLAIKCM LTAKVEKKDQ DKFYNSIVYG IAPPPQFKKR
65 75 85 NSP1 95 105 115
YNTSDNSRGM NYETIMFNKV AVLICEALNS IKITQSDVAN VLSRVVSVRH LENLVLRKEN
125 135 145 155 165 175
HQDVLFHSKE LLLKSVLIAI GQSKEIETTA TAEGGEIVFQ NAAFTMWKLT YLDHKLMPIL
Loop
185 195 205 215 225 235
DQNFIEYKIT LNEDKPISDV CIKELVAELR WQYNRFAVIT HGKGHYRVVK YSSVANHADR
245 255 265 275 285 295
NSP4/VP3
NSP5
VFATYKNSAK SGNVIDFNLL DQRIIWQNWY AFTSSMKQGN TLDVCKKLLF QKMKQEKNPF
305 315
KGLSTDRKMD EVSHVGI
Green: N-terminal domain
Red: C-terminal domain
Domain 1
Domain 2
Internal loop
Beta strand 7
Beta strand 9
Internal loop
N-side of the cleft
C-side of the cleft
NTPase acitivity

I

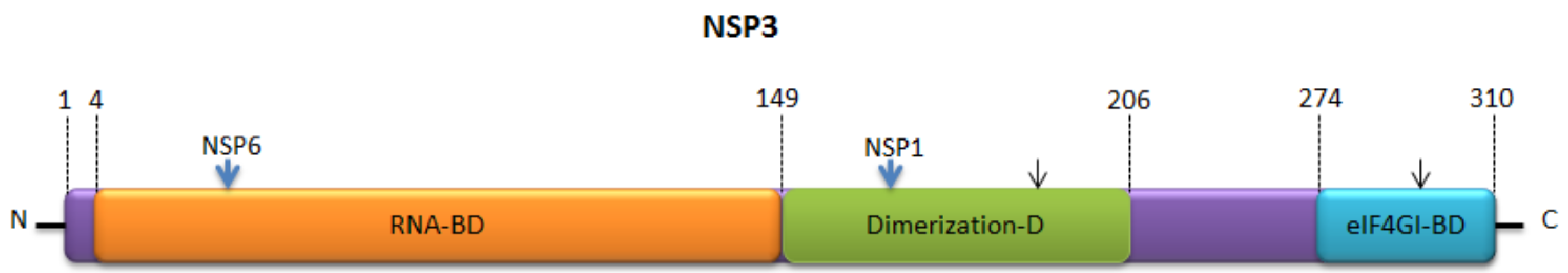

RNA-BD: RNA binding domain; **Dimerization-D**: Dimerization domain; **eIF4G-BD**: Eukaryotic translation initiation factor 4 G binding domain.

J

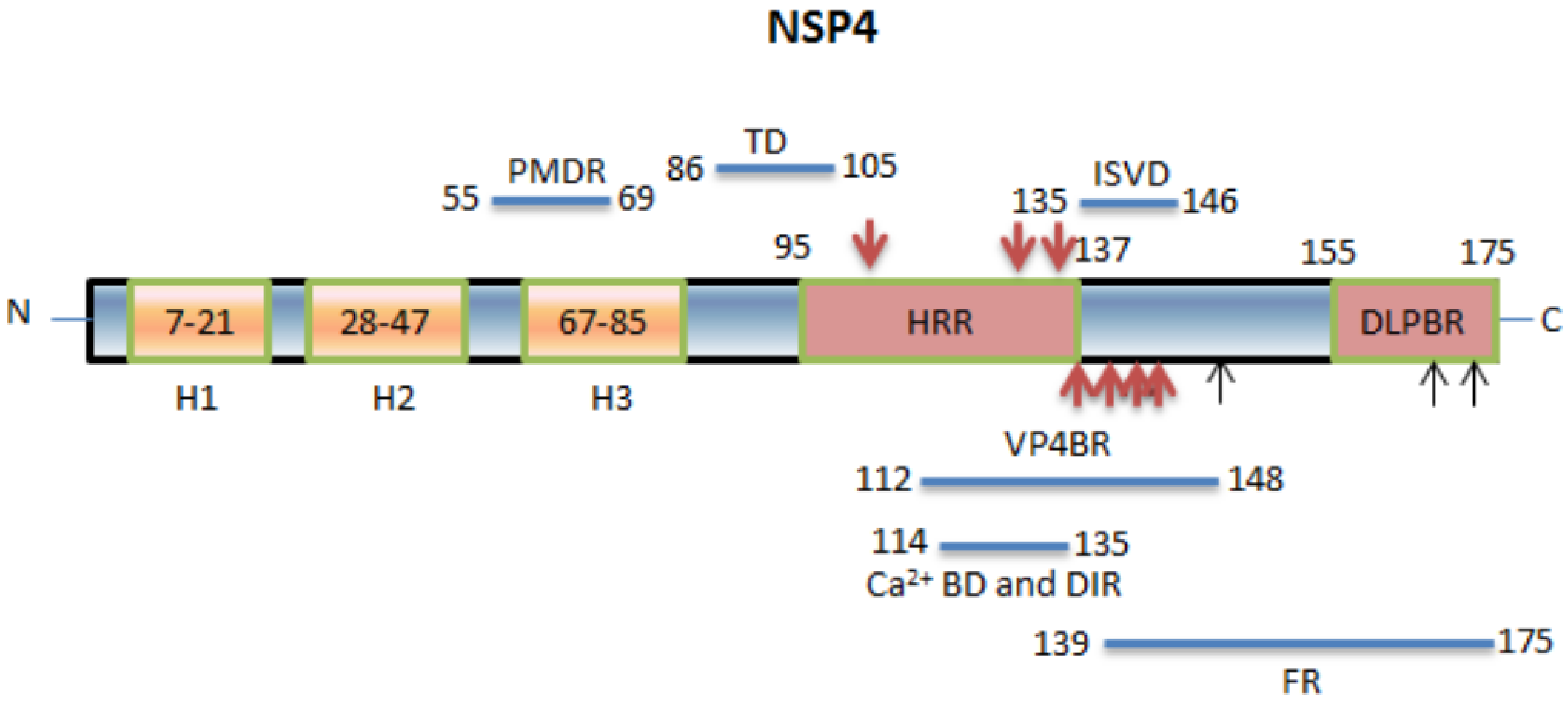

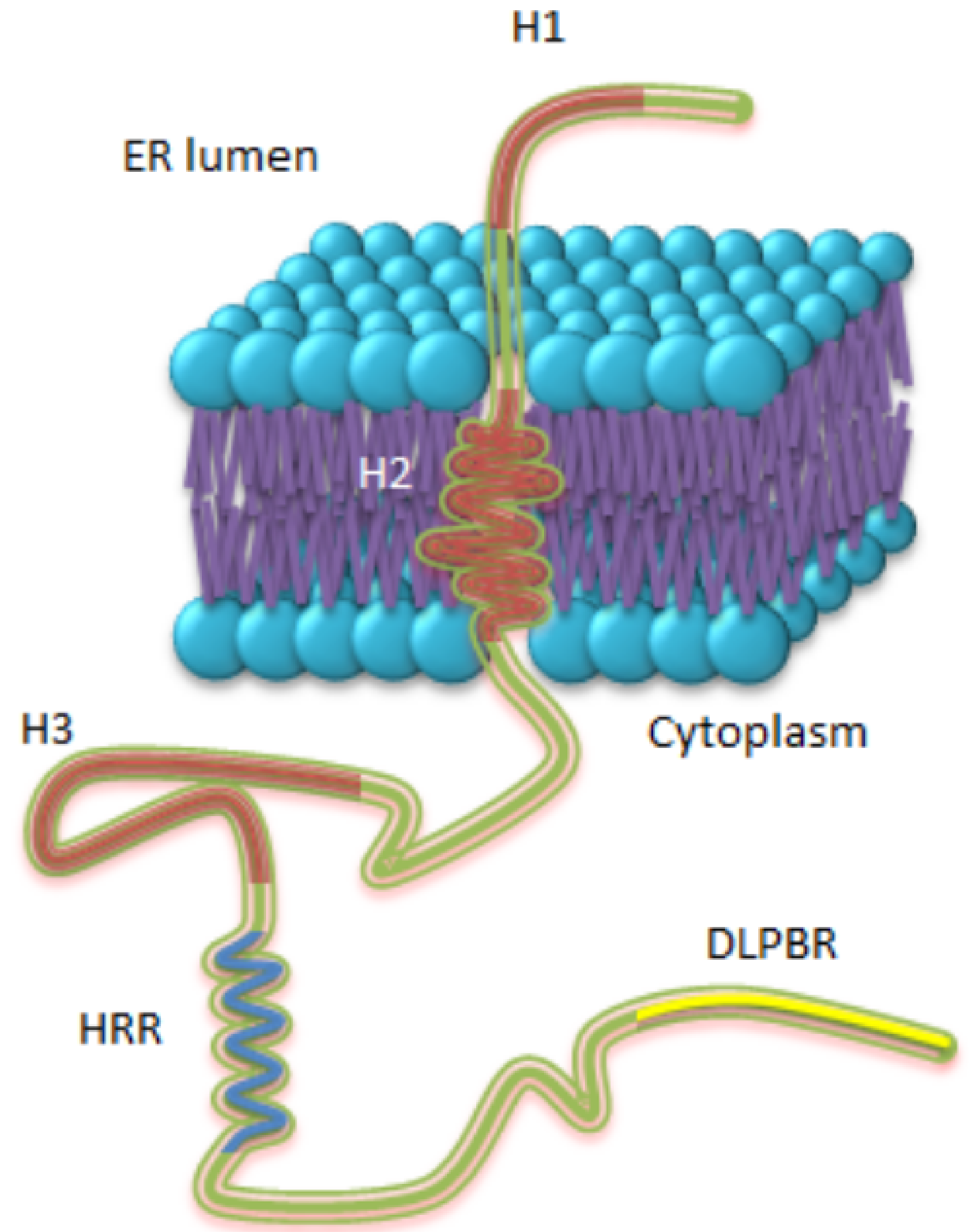

H1, H2 and H3: hydrophobic domains**; PMDR:** proximal membrane-destabilizing region; **TD**: tetramerization domain; **HRR**: heptad repeat region; **VP4BR**: VP4-binding region; **Ca^{2+} BD and DIR**: calcium-binding and diarrhea-inducing region; **ISVD** interspecies variable domain; **FR:** flexible region; **DLPBR** double-layered particle-binding region; Inter-coevolving sites are shown by red arrows whereas the intra-coevolving sites are shown by thin black arrows.

K

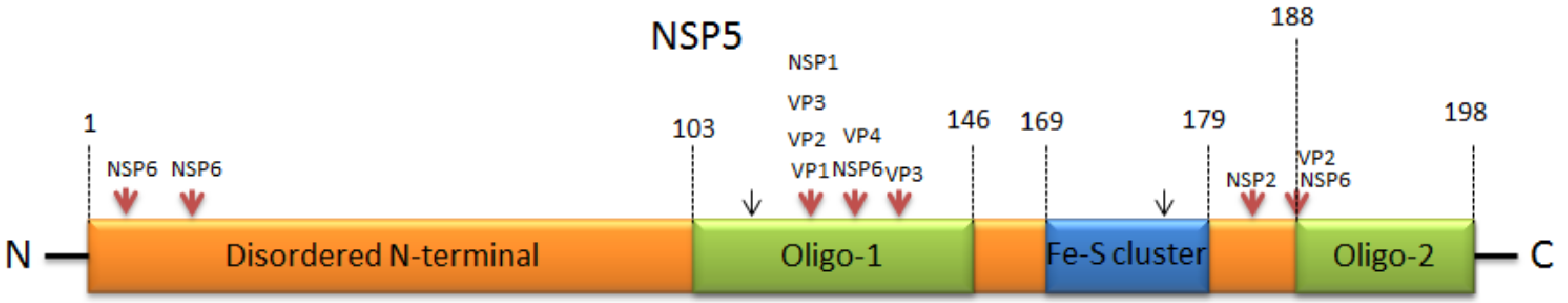

Supplement: Supplementary file 1 [file genes-11-00028-s001.zip › Figure S5.pdf]
