# Supplementary figures and images for: Coding-Gene Coevolution Analysis of Rotavirus Proteins: A Bioinformatics and Statistical Approach
Source: Genes (Basel). 2019 Dec 24;11(1):28. doi: 10.3390/genes11010028 (PMC7016848; doi:10.3390/genes11010028)

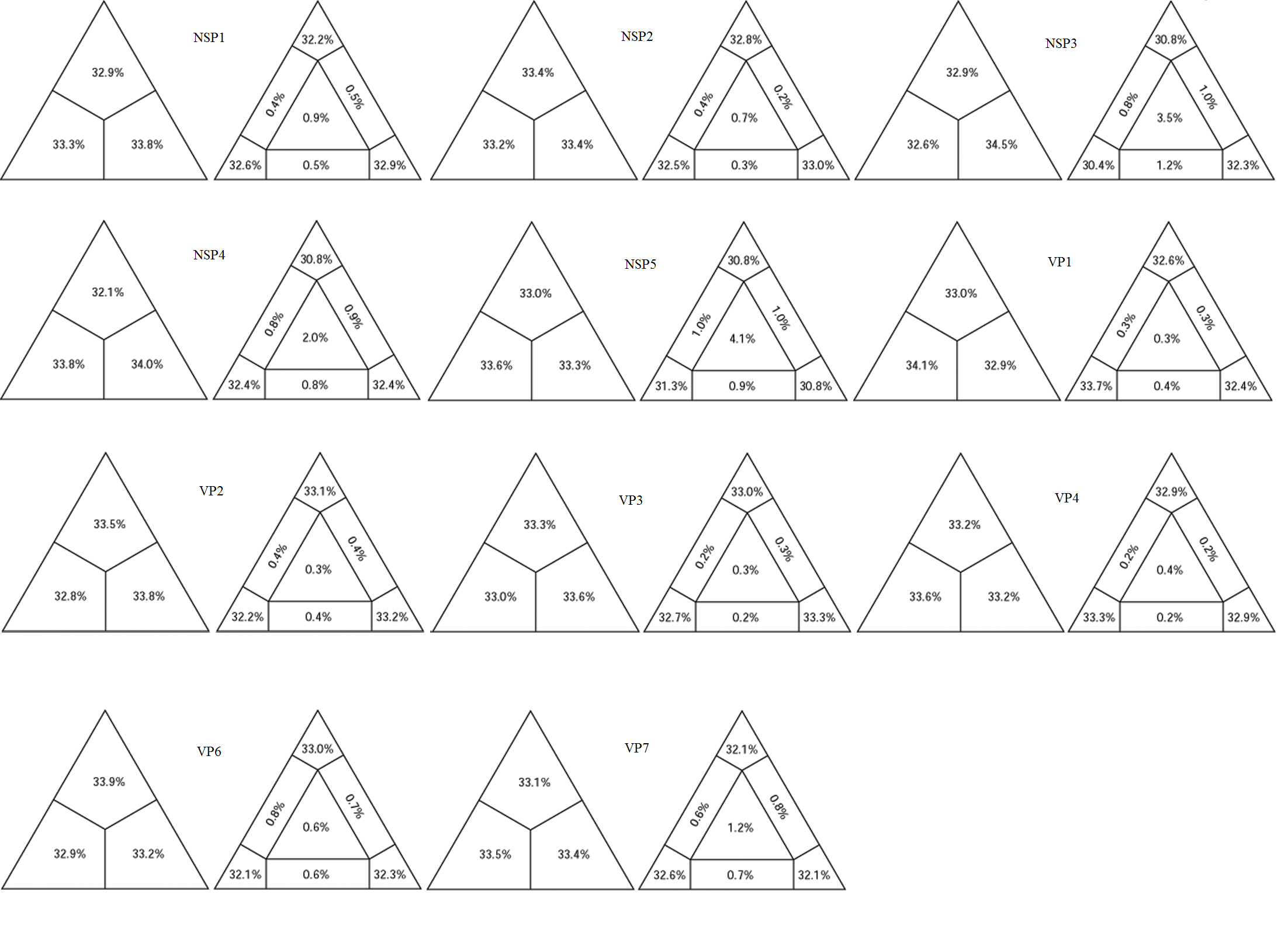

Supplement: Supplementary file 1 [file genes-11-00028-s001.zip › Figure S1.png]

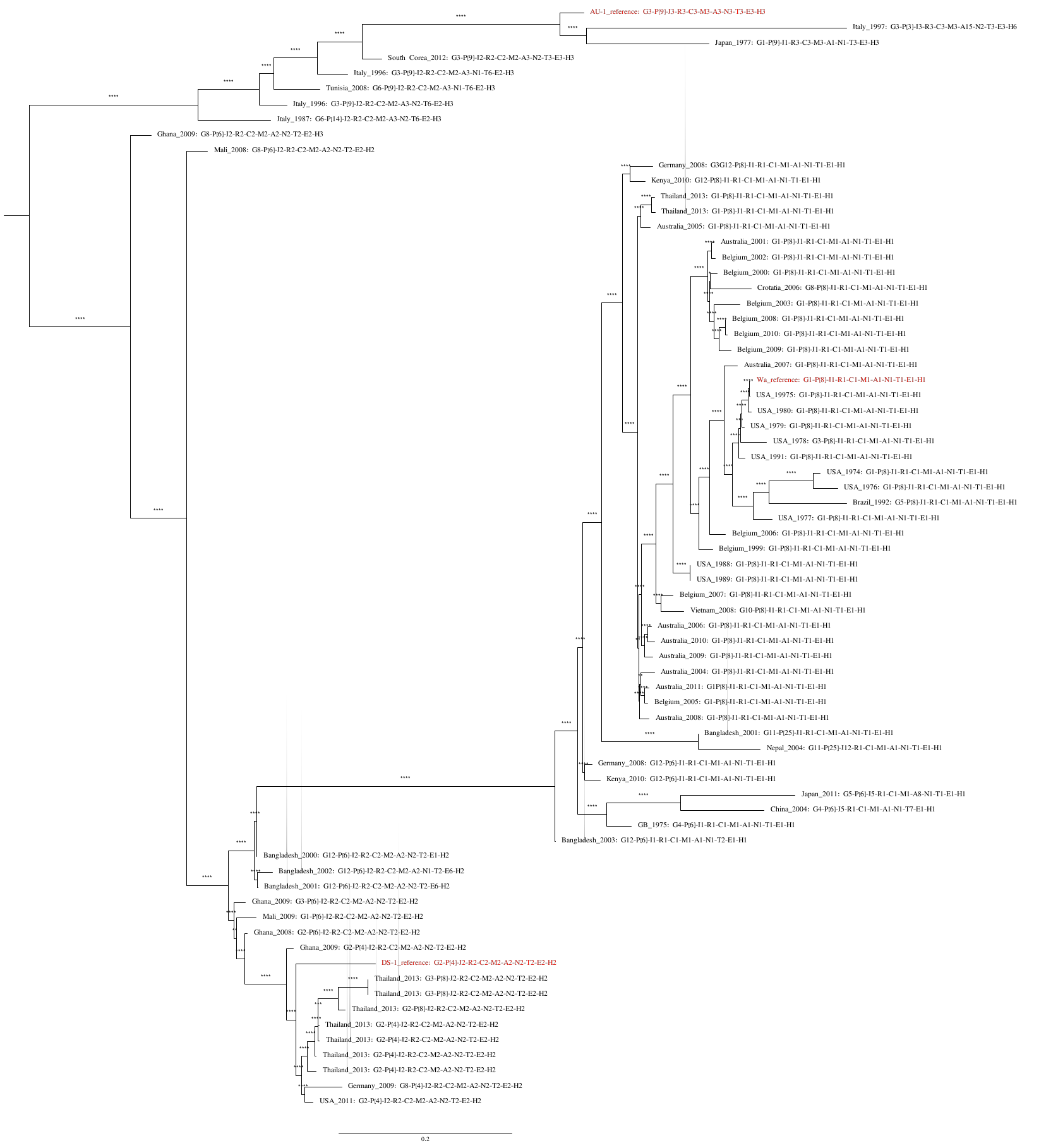

Supplement: Supplementary file 1 [file genes-11-00028-s001.zip › Figure S2.png]

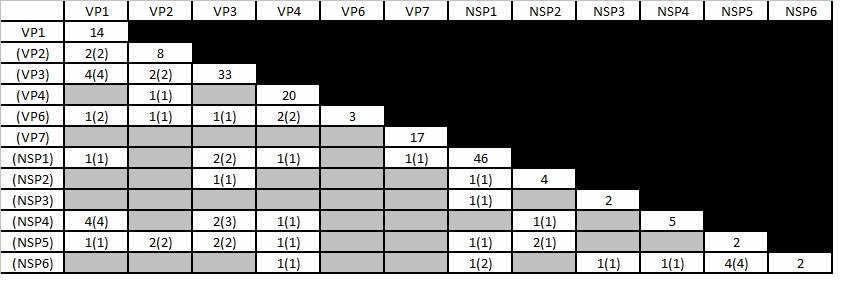

Supplement: Supplementary file 1 [file genes-11-00028-s001.zip › Figure S3.png]

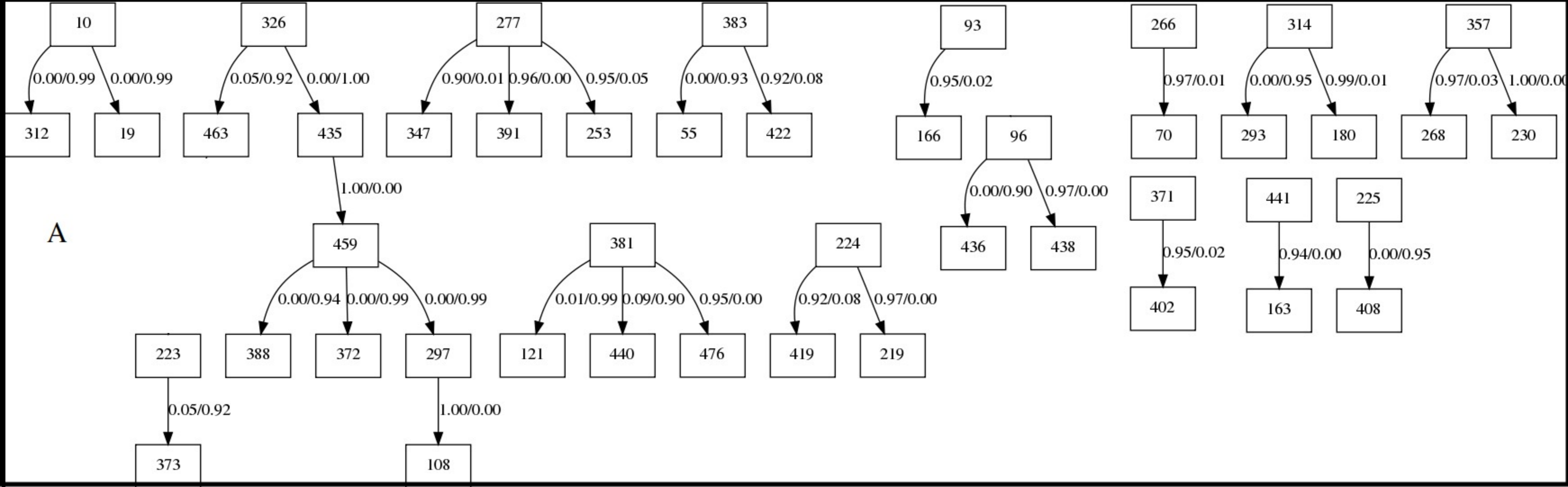

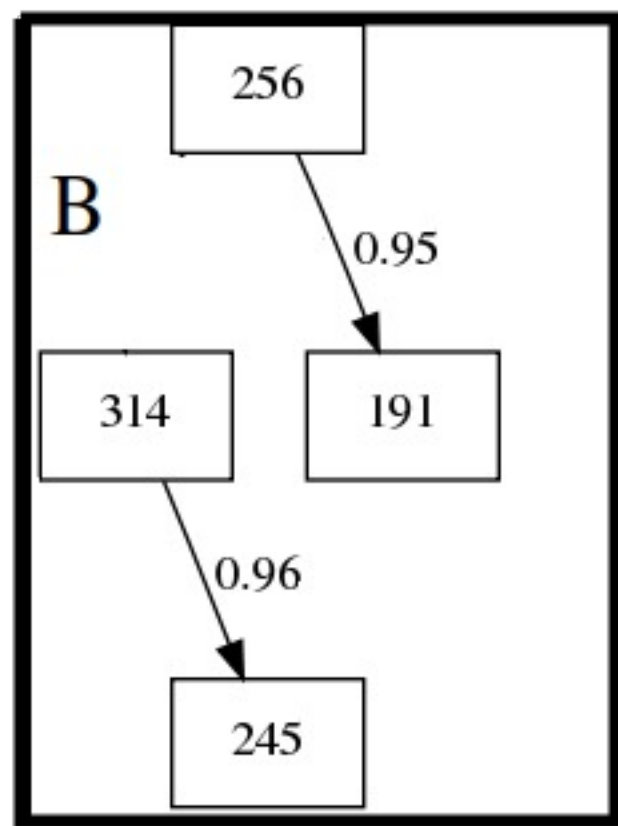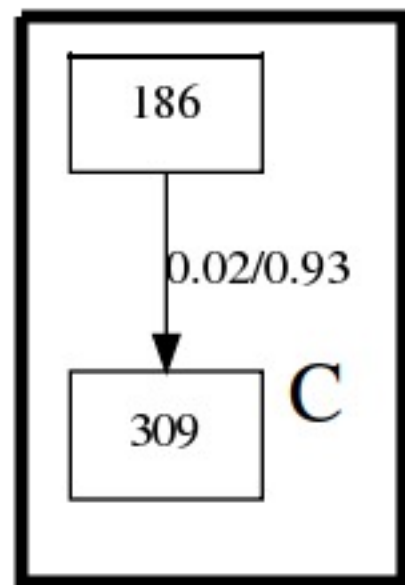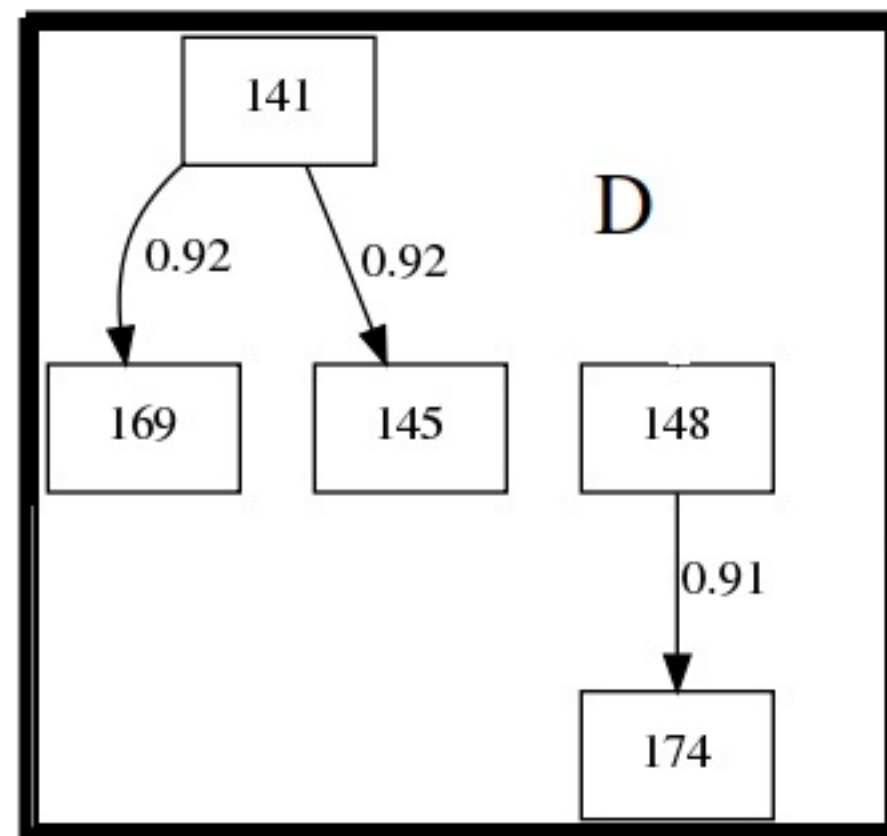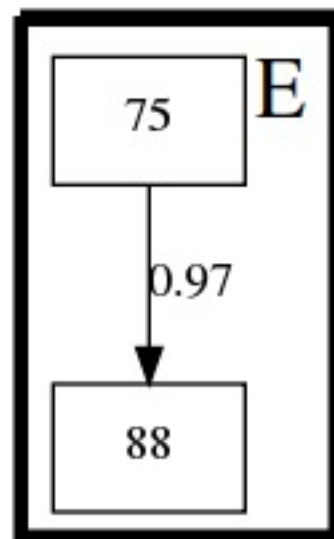

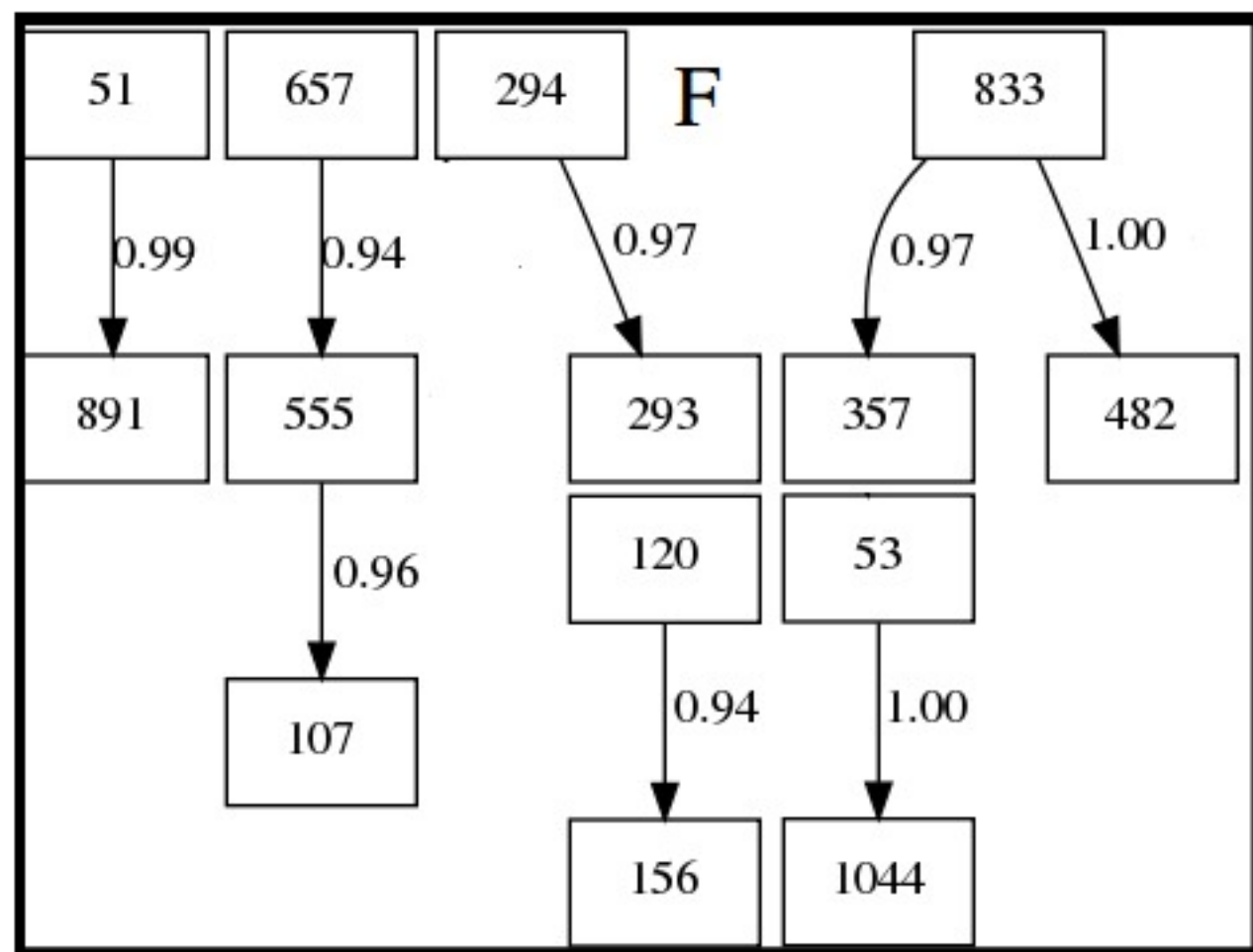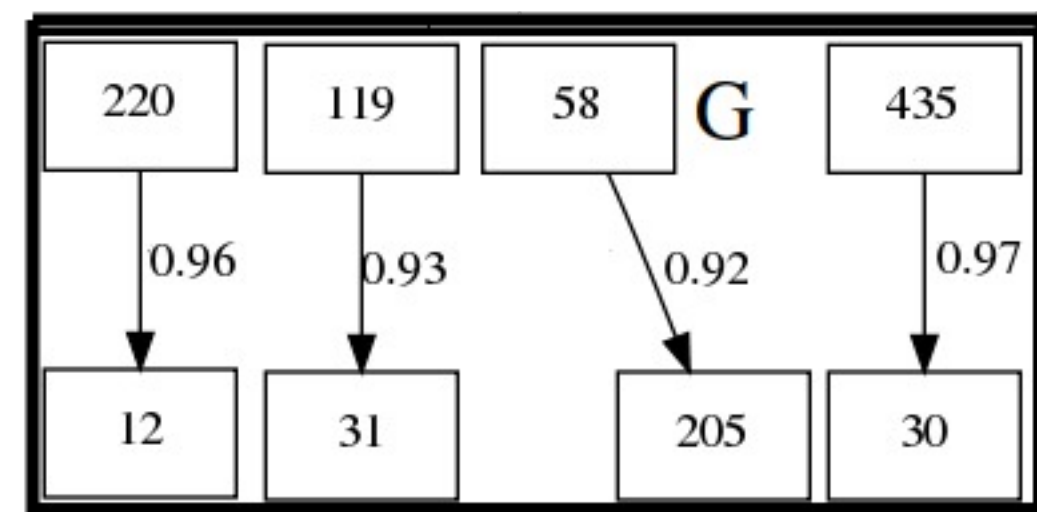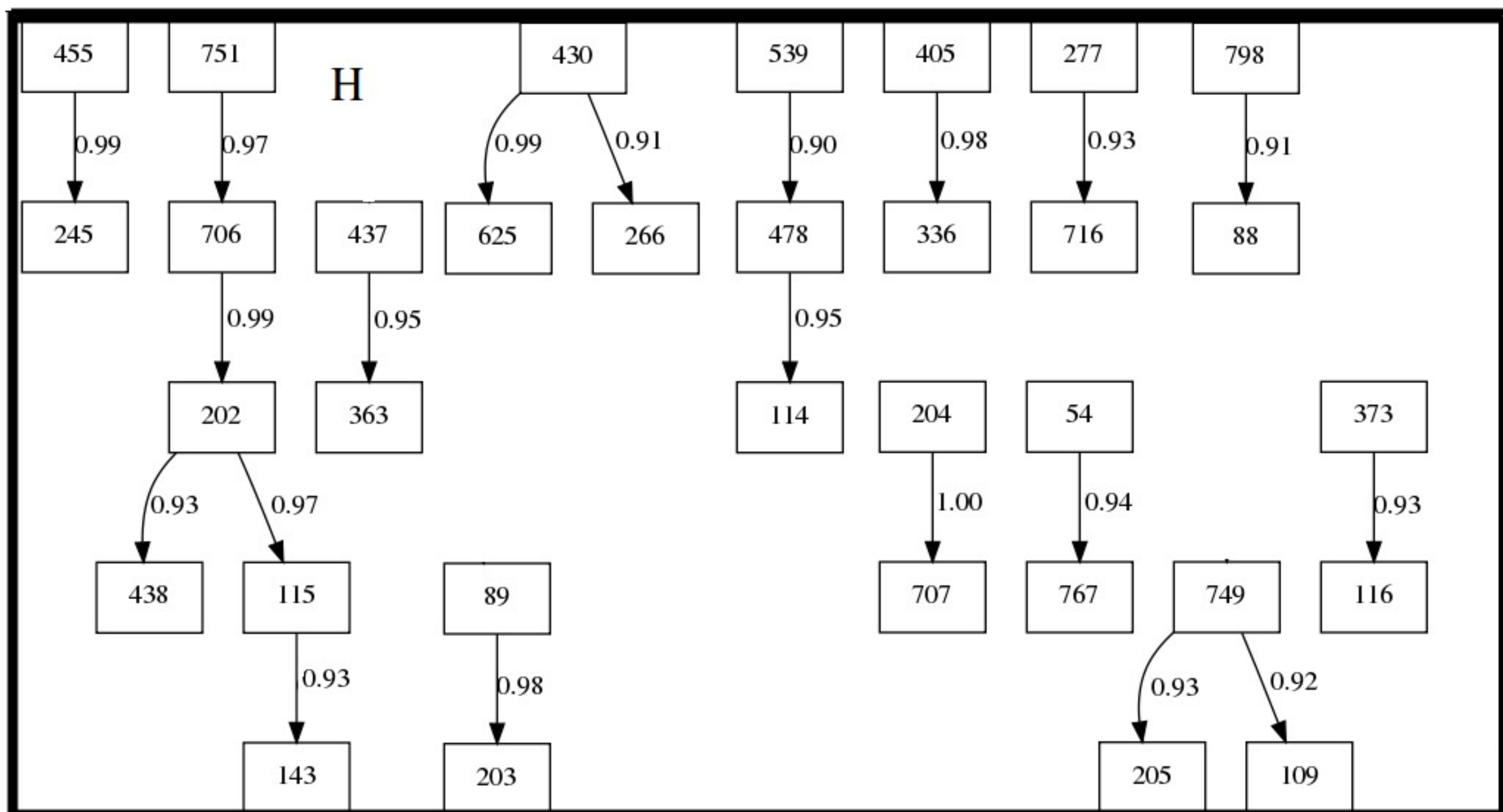

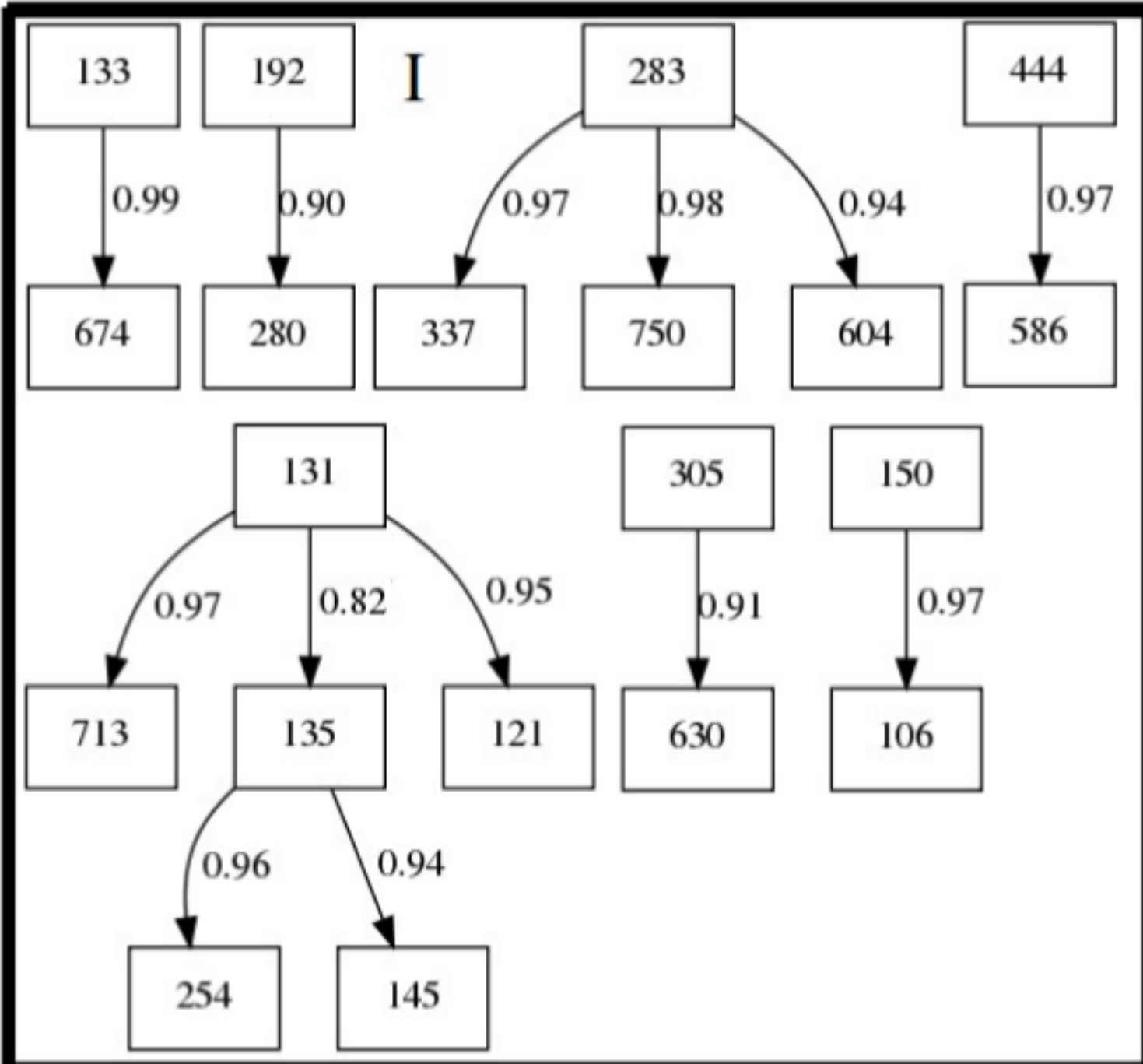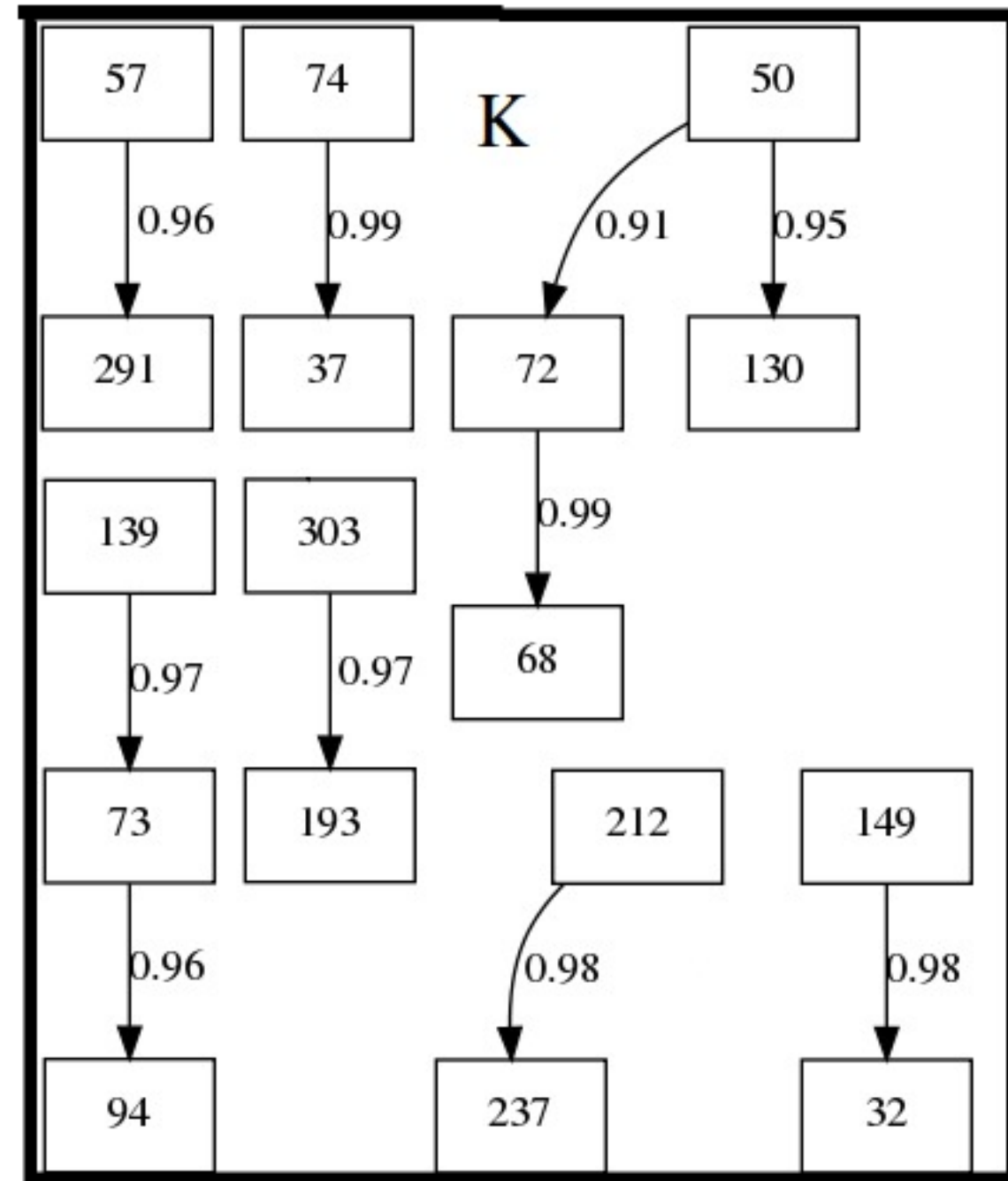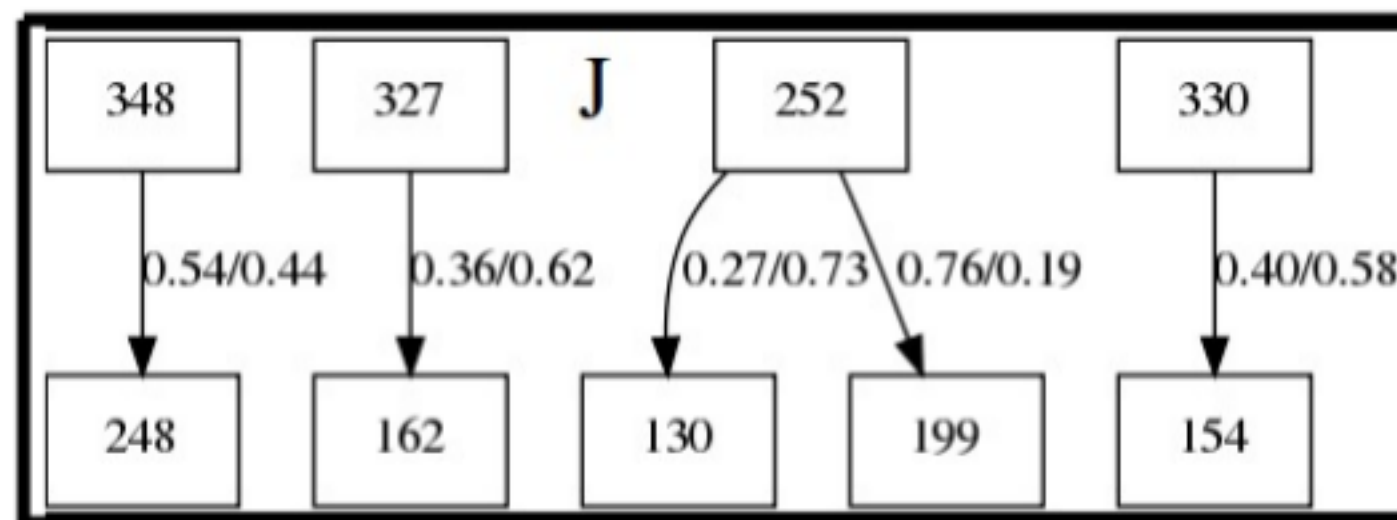

Supplement: Supplementary file 1 [file genes-11-00028-s001.zip › Figure S4.pdf]

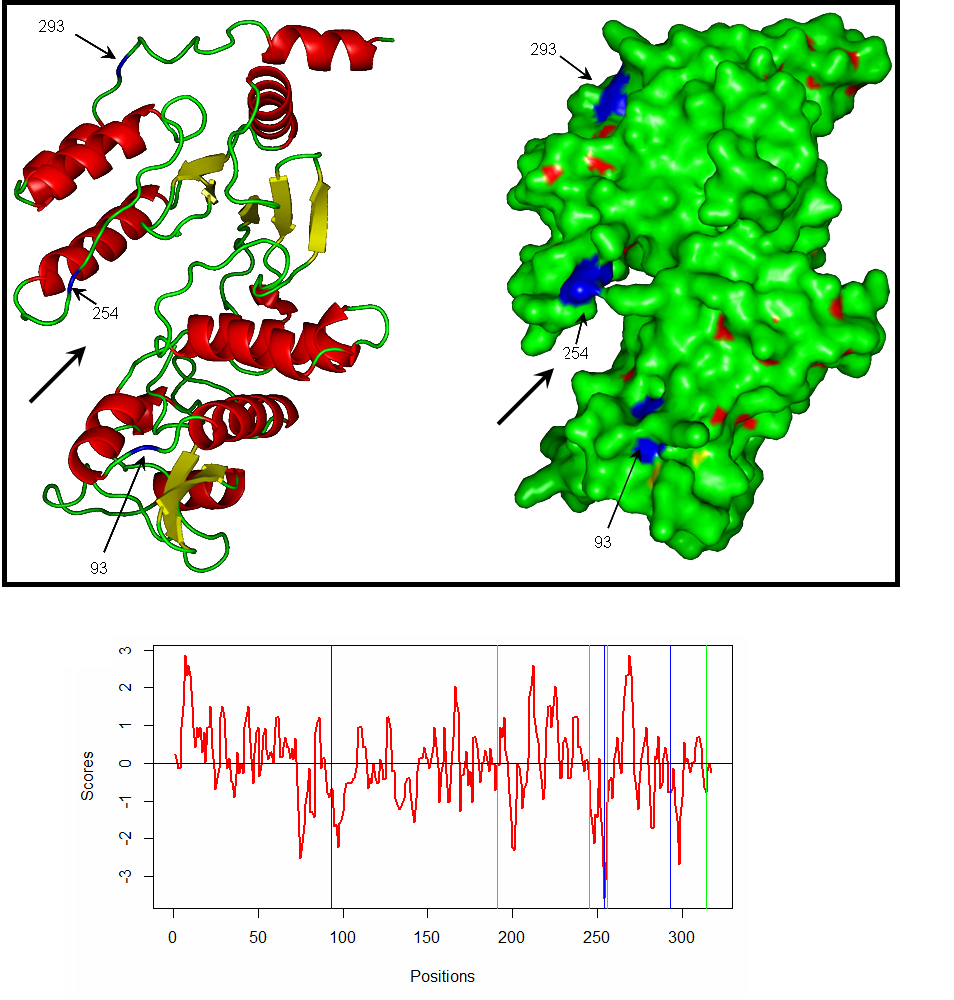

Supplement: Supplementary file 1 [file genes-11-00028-s001.zip › Figure S6.png]

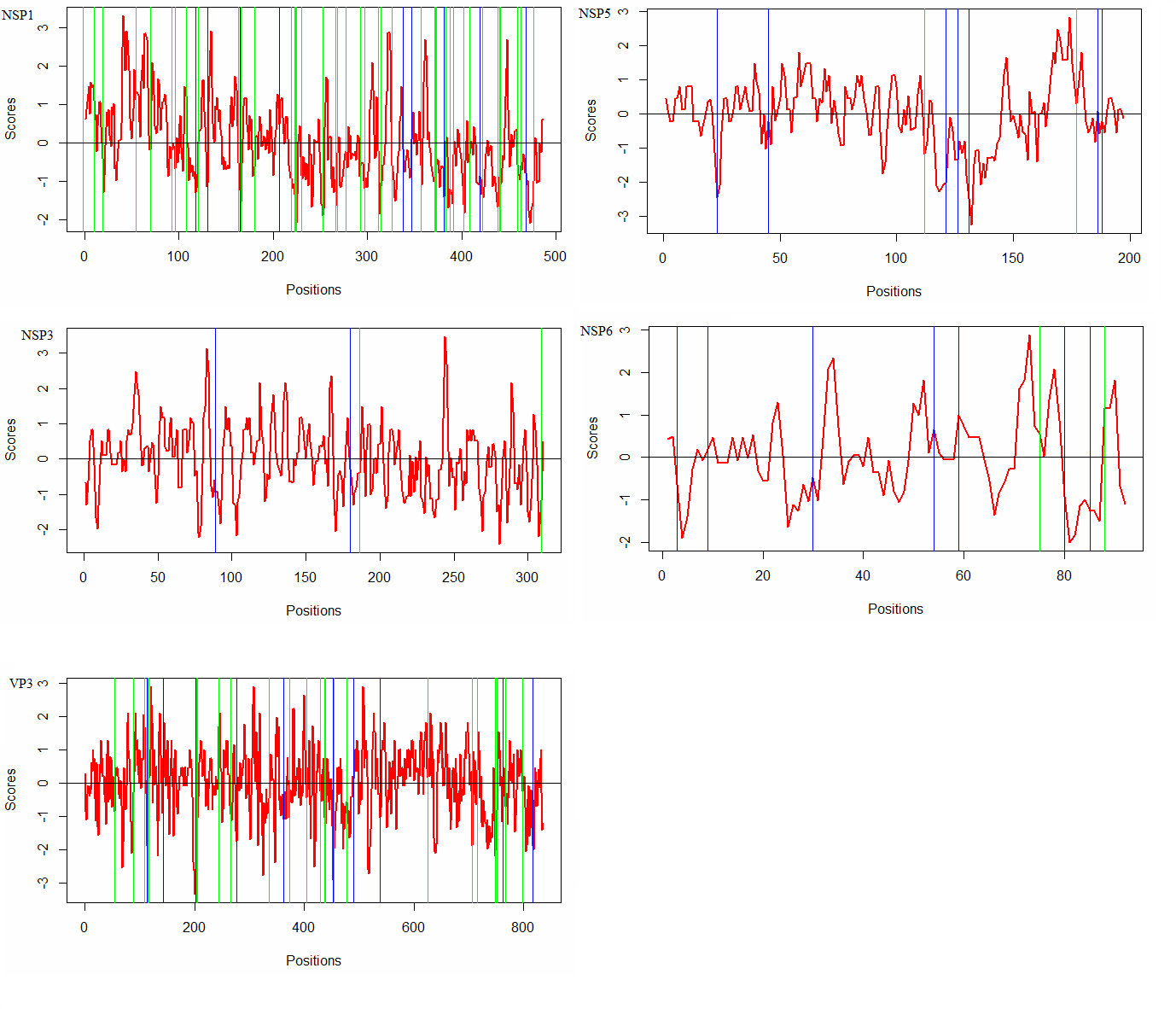

Supplement: Supplementary file 1 [file genes-11-00028-s001.zip › Figure S7.png]

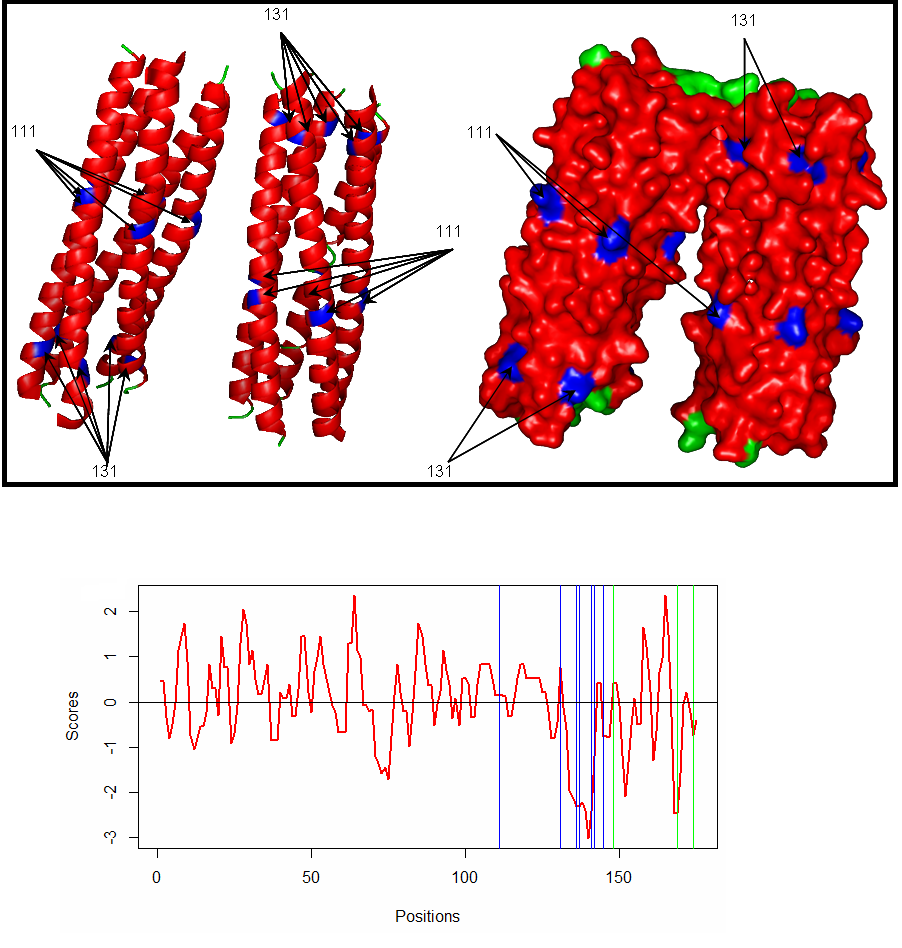

Supplement: Supplementary file 1 [file genes-11-00028-s001.zip › Figure S8.png]

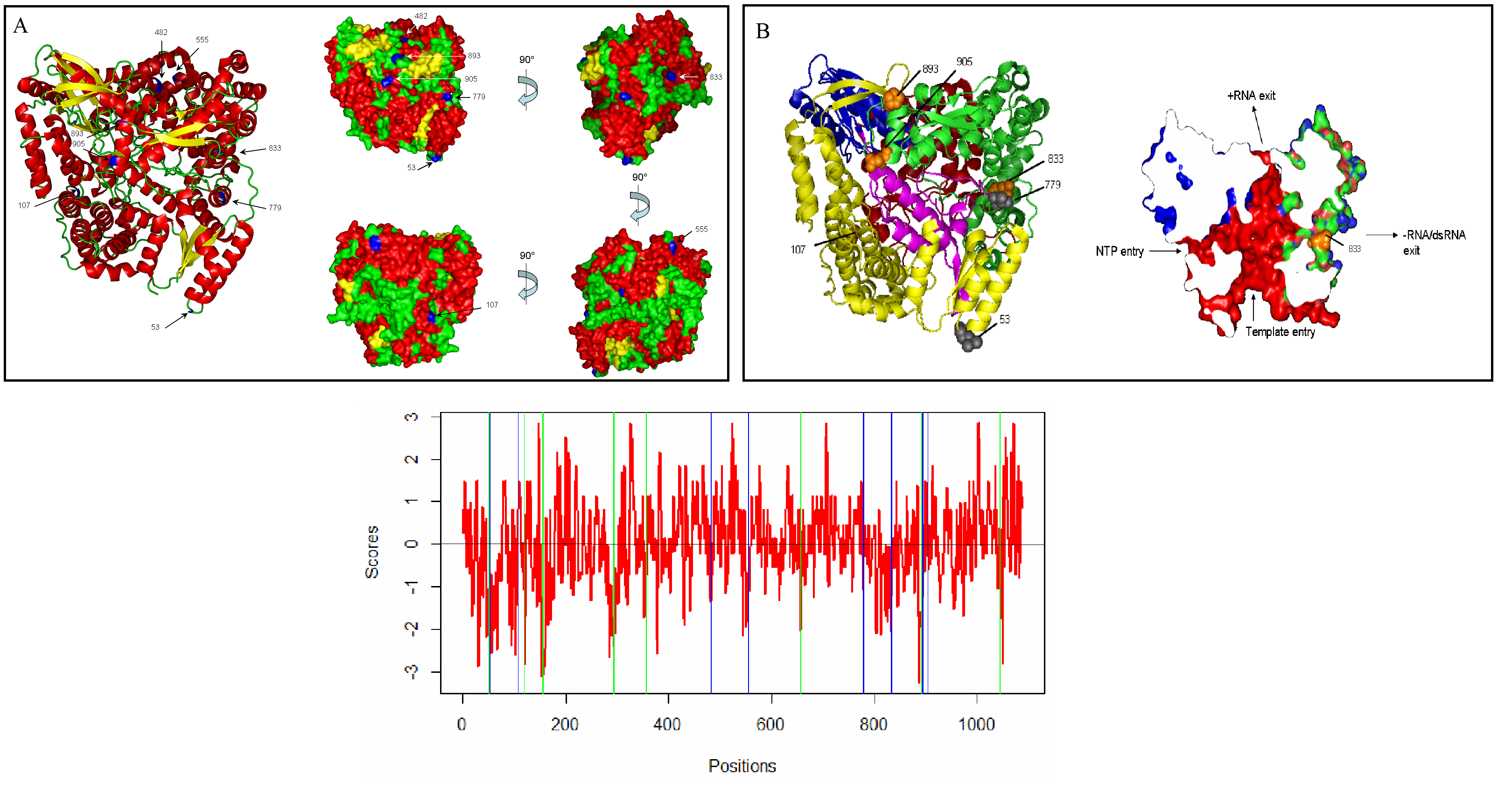

Supplement: Supplementary file 1 [file genes-11-00028-s001.zip › Figure S9.png]

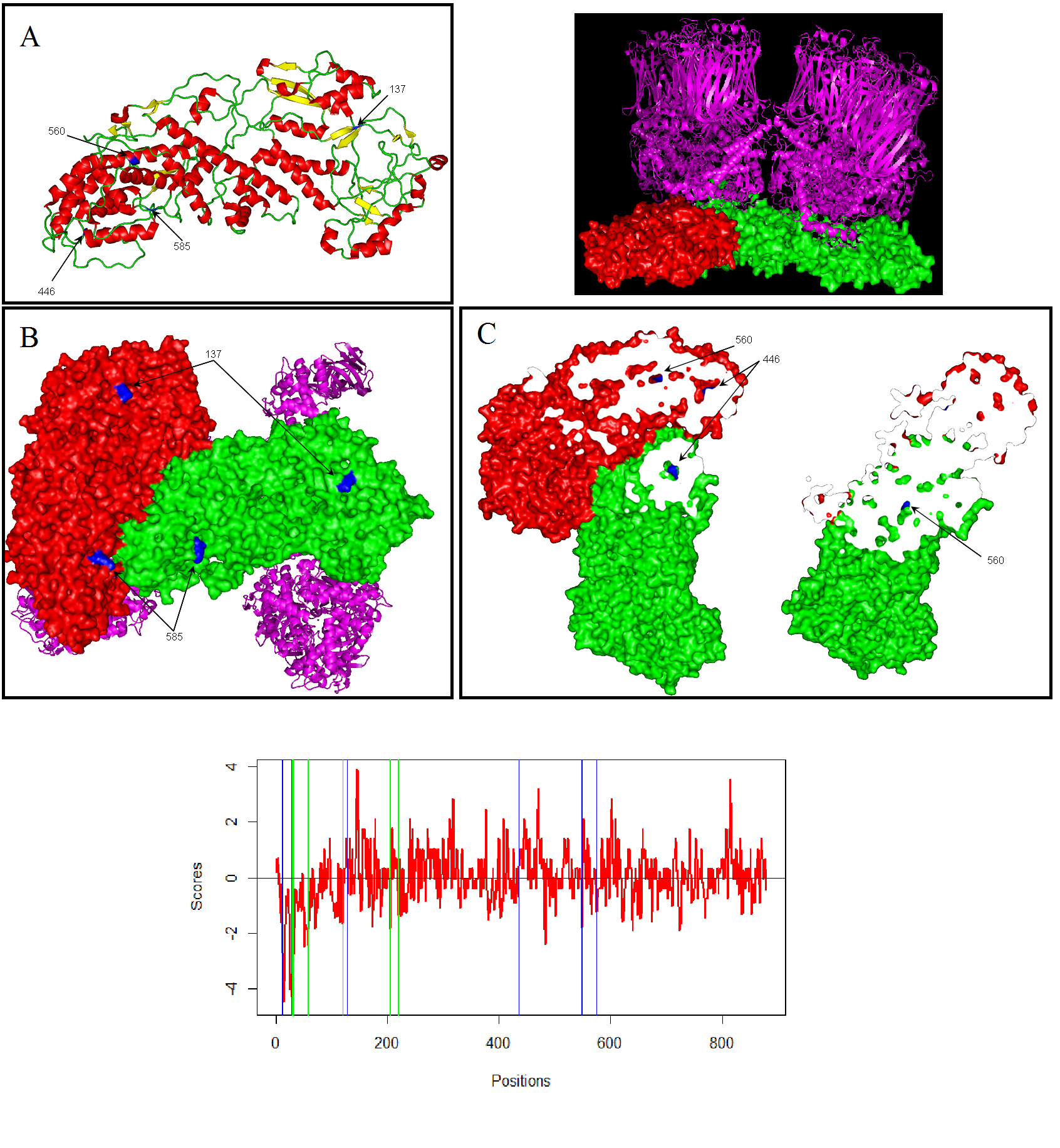

Supplement: Supplementary file 1 [file genes-11-00028-s001.zip › Figure S10.png]

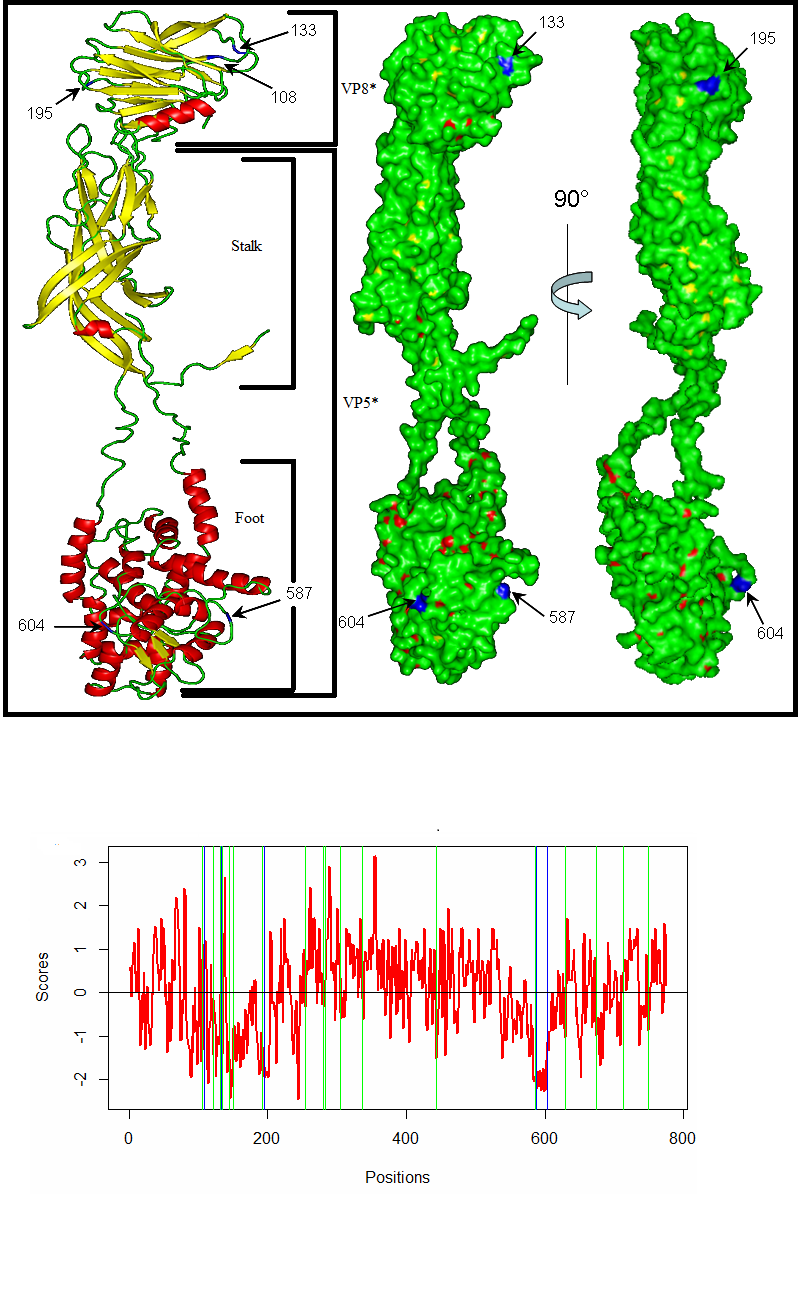

Supplement: Supplementary file 1 [file genes-11-00028-s001.zip › Figure S11.png]

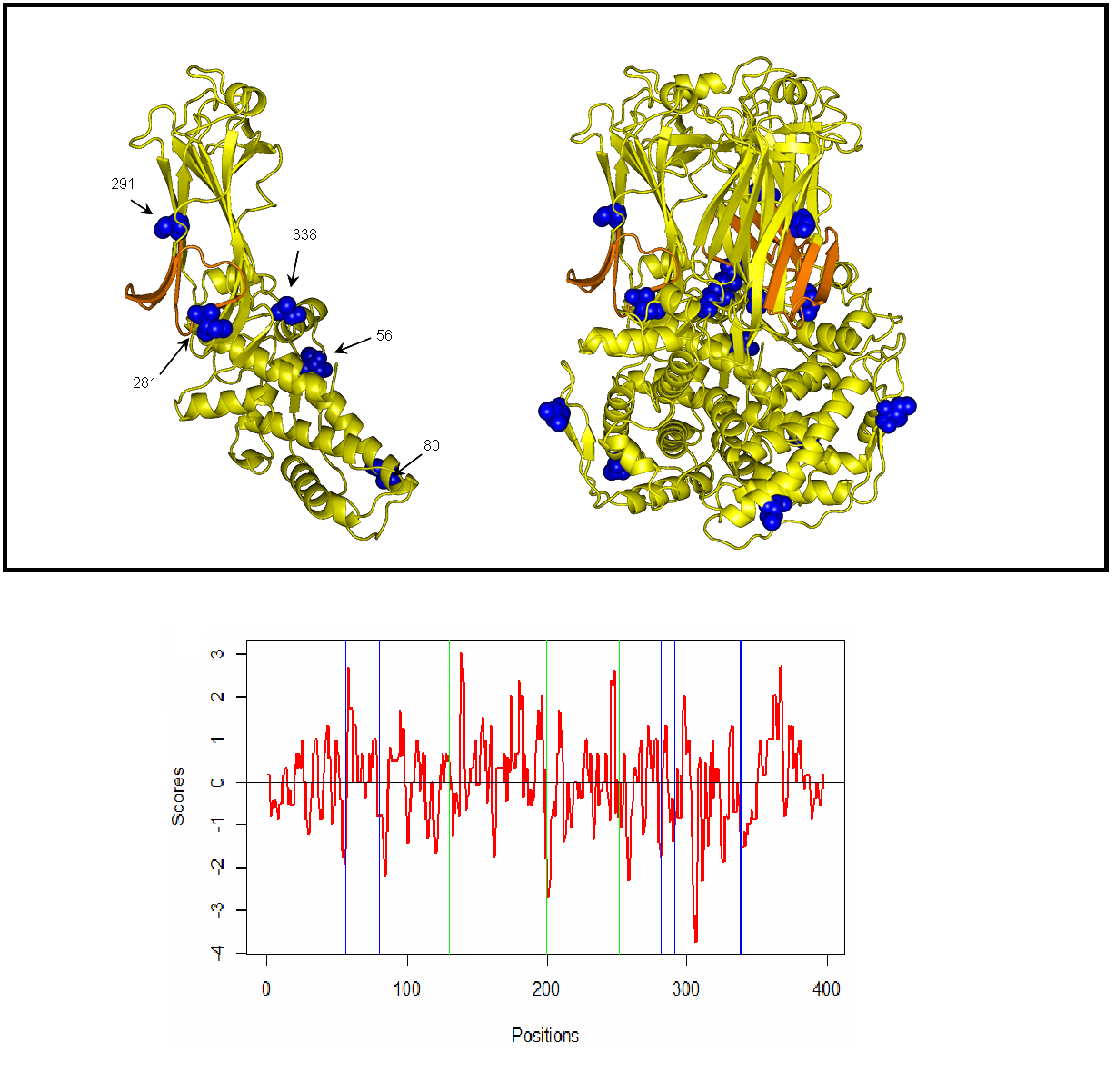

Supplement: Supplementary file 1 [file genes-11-00028-s001.zip › Figure S12.png]

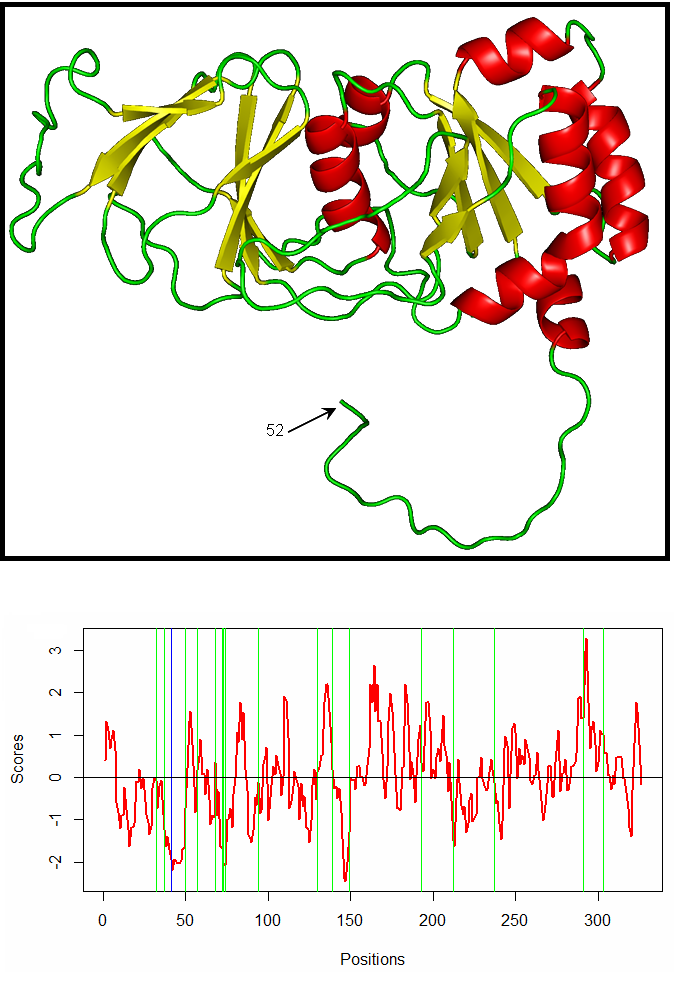

Supplement: Supplementary file 1 [file genes-11-00028-s001.zip › Figure S13.png]

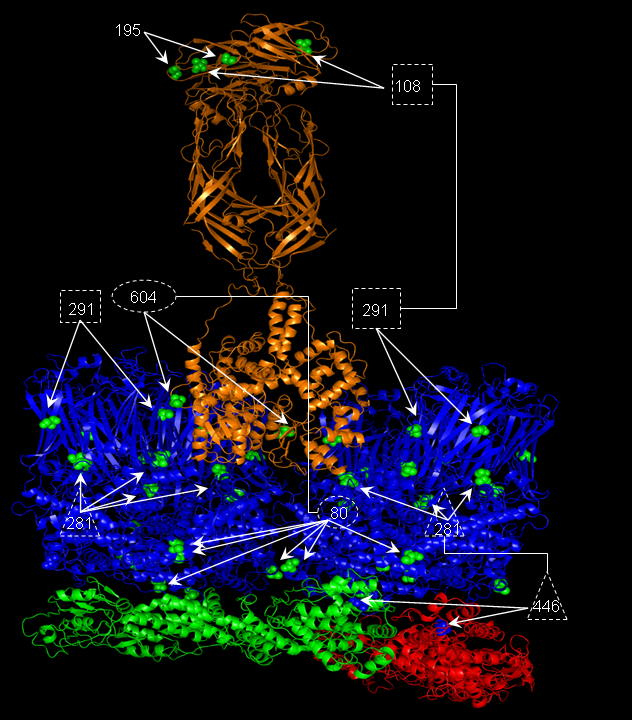

Supplement: Supplementary file 1 [file genes-11-00028-s001.zip › Figure S14.png]

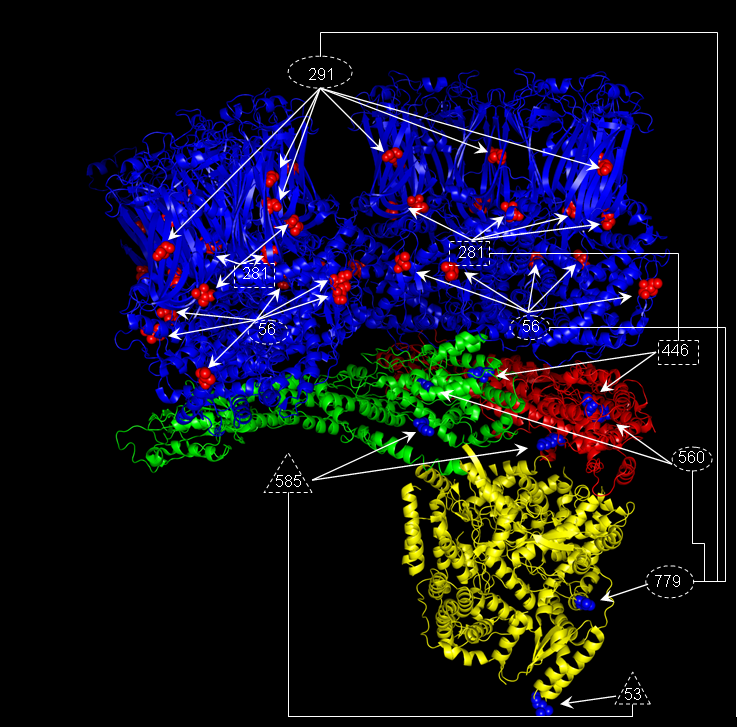

Supplement: Supplementary file 1 [file genes-11-00028-s001.zip › Figure S15.png]
